# A community-guided approach to monitoring contaminants of emerging concern in freshwater systems using passive samplers

**DOI:** 10.1038/s44454-025-00021-1

**Published:** 2026-01-07

**Authors:** Alexandra K. Richardson, Stav Friedman, Leon P. Barron

**Affiliations:** MRC Centre for Environment & Health, Environmental Research Group, School of Public Health, Faculty of Medicine, Imperial College London, London, UK

**Keywords:** Ecology, Ecology, Environmental sciences, Hydrology

## Abstract

Chemical pollution in the aquatic environment is a global issue, representing a threat to human and environmental health, especially for unregulated contaminants of emerging concern (CECs). Identifying and prioritising pollution sources requires high spatio-temporal resolution data and strategic site selection; here, local knowledge provides valuable insight. This study evaluated a community-guided approach for monitoring CECs in freshwater systems using a miniaturised 3D-printed passive sampler device (3D-PSD). Seventeen citizen scientists from Sheffield, Norwich, and London (England) selected sites along their local rivers and were trained to deploy 3D-PSDs and collect water samples. A total of 51 CECs were quantified in the water and passive sampler extracts, with ketamine, lidocaine, tramadol, and venlafaxine occurring in over 50% of samples. All compounds were present below the medium-risk threshold. Results were shared via an online meeting and a public-facing report. Participants highlighted the importance of rivers and other blue-green spaces in their daily lives and reported spending up to four hours at their local rivers per visit. They identified key areas for improving the field deployment procedure and the data collection system. This work demonstrates the applicability of the 3D-PSD to large-scale citizen science and other community engagement projects for freshwater monitoring.

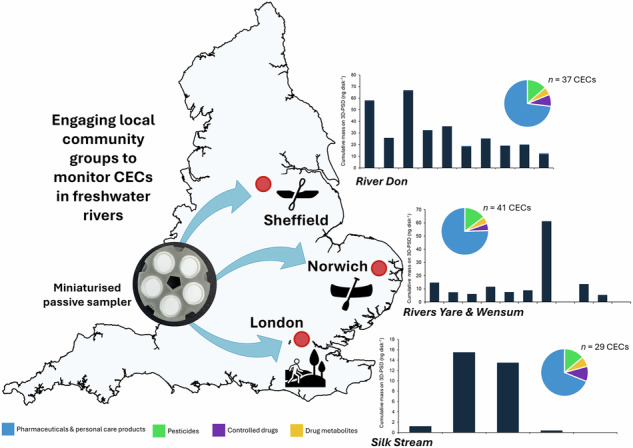

## Introduction

Large-scale monitoring of chemical contaminants in the environment represents a significant challenge, both logistically and analytically. Over 219 million chemicals are registered in the Chemical Abstracts Service (CAS) database, and it is estimated that there are up to 10^60^ possible unique organic structures under 500 Da that exist within the chemical space^1^. A very small subset of these can be measured using existing analytical techniques. Monitoring chemical contamination in rivers globally has identified over 630 unique contaminants using conventional analytical techniques, such as liquid chromatography (LC) or gas chromatography (GC) coupled to mass spectrometry (MS) for targeted compound lists^2–6^. The emergence of high-resolution accurate mass spectrometry (HRMS) in suspect screening and/or non-target modes can aid in the identification of unknowns^7^. The detection and identification of unknowns in a sample using analytical instruments represent the final step in the analysis pipeline. The initial steps of site selection and sample collection are often viewed as relatively straightforward processes, but can unexpectedly present logistical and practical challenges, especially when required at a large spatio-temporal scale.

To reliably capture the impact and magnitude of chemical pollution in water systems, one of the most important considerations is how, where, and when to take the sample for analysis. Grab sampling is a relatively straightforward sampling technique that is useful for capturing and monitoring short-term elevated contaminant exposures; however, this technique is laborious and logistically intensive when used to monitor chronic exposure at a high spatio-temporal scale. Alternative approaches to monitor chronic exposures include real-time sensing^8^, automated composite sampling^9^, and passive sampling. The location of a sample must be considered in the context of other samples collected from the same water system to identify sources of contamination (diffuse and/or point sources). As an example, discharges from wastewater treatment works or combined sewage overflow points (CSOs) are usually characterised by a significant increase in the number, type and concentration of specific contaminants of emerging concern (CECs) compared to other water samples collected from the river catchment during the same campaign^10,11^. Taking this information further, computational models have recently been used to apportion sources of chemicals using data collected through monitoring campaigns using grab water samples^12^. To maximise knowledge and identify new pollution sources, high spatio-temporal resolution water data need to be collected, analysed, and key sites identified for continued monitoring. This initial pilot work, which is undoubtedly necessary, can be costly and laborious. Utilising knowledge from local communities to identify the potential occurrence and sources of pollution, and to scale up monitoring capability can be invaluable. Engaging the local communities in scientific research also raises awareness of environmental issues, management challenges, how individuals contribute to the issue, and empowering them to develop strategies to minimise their impact^13^.

The term ‘citizen science’ is broadly used to define any public participation in scientific research and knowledge generation and has successfully been utilised in various water quality monitoring studies due to the larger spatial and temporal scales at which data can be collected^14,15^. In addition, citizen science is an important tool for engaging the public in scientific research^16^. To date, citizen science has predominantly been used to collect data on general water quality parameters such as physical (temperature and conductivity^17^), chemical (pH and nitrate/phosphate concentrations^18^), and biological (macroinvertebrate species characterisation^19^ and *Escherichia coli* counts^20^) conditions. These parameters are most frequently tested due to the ease of equipment use, low training requirements and the low cost of testing kits^15^. By comparison, traditional CEC monitoring using citizen science is generally under-represented in the scientific literature due to challenges with analyte stability and transporting large volumes of water samples (~ 1 L) from the collection site to the analytical laboratory for analysis^21,22^.

We recently developed a 3D-printed passive sampler device (3D-PSD) for the monitoring of CECs in river water^23^. Due to its small size, ease of use, and low cost, this device could potentially be utilised by citizen scientists in large numbers for river water monitoring programmes. Passive samplers have previously been used in published citizen science projects, mainly to measure air quality (e.g., nitrogen dioxide (NO_2_) in urban environments^24,25^, volatile organic compounds (VOCs) and polycyclic aromatic hydrocarbons (PAHs) in indoor environments^25^, and the occurrence of air-born *Aspergillus fumigatus* in the environment^26^), but have not been used as extensively to measure water quality.

The aim of this work was to collaborate with citizen scientists to monitor English freshwater rivers for CECs using 3D-PSDs. This was achieved through the following objectives: a) to identify and engage local river users in three English cities to take part in the study; b) to deliver training workshops to the citizen scientists to teach where and how to deploy, collect, and return the 3D-PSD; c) to analyse the returned 3D-PSDs for 164 CECs using a rapid targeted LC-MS/MS method; and d) present the findings to the citizen scientists and collect feedback about the project. Therefore, this work represents one of the first studies to trial a miniaturised passive sampler device for use by citizen scientists to measure CECs in freshwater.

## Results

### Occurrence of contaminants in river water

**Fig. 1:**
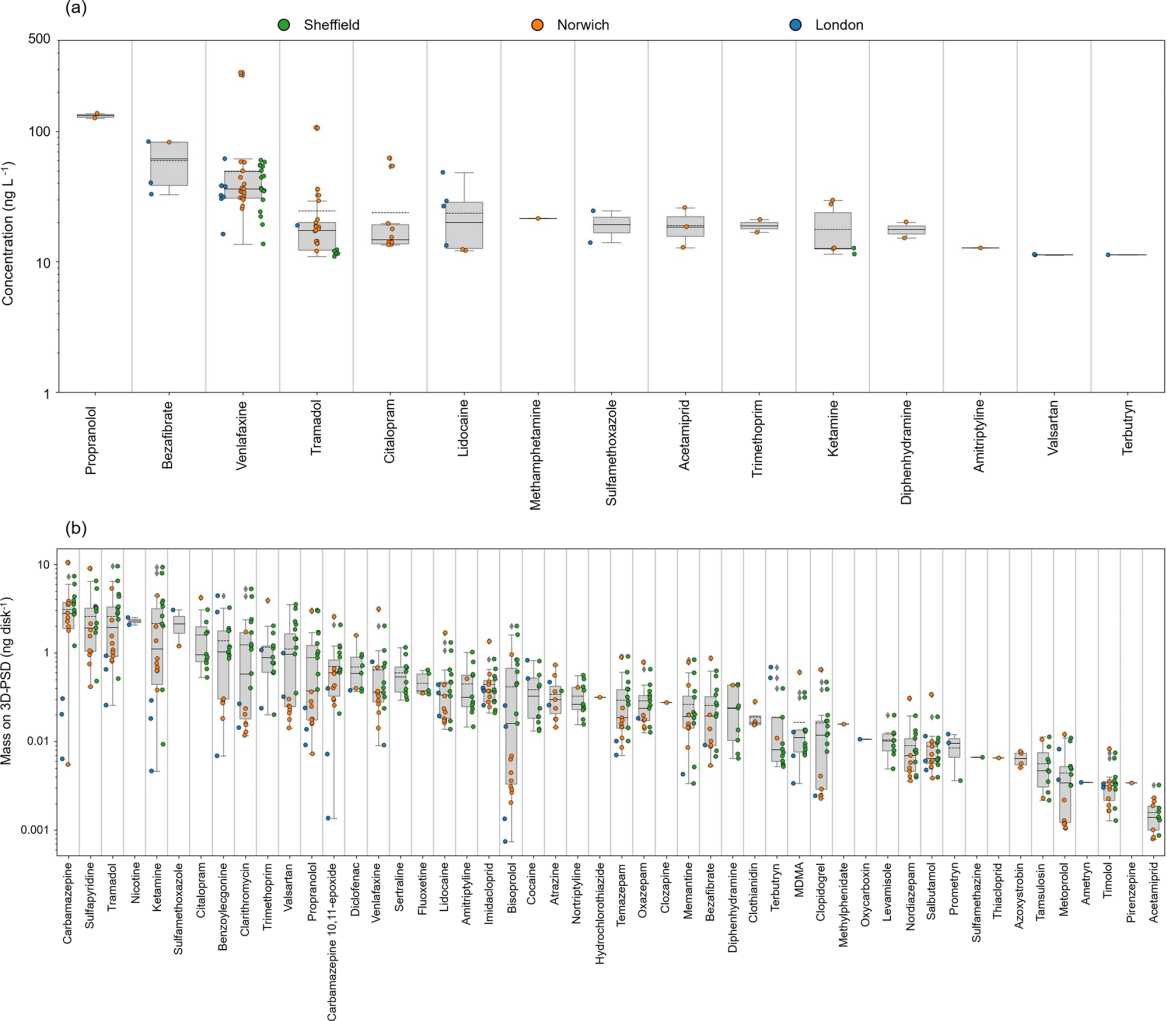
Contaminants measured in water and using the 3D-PSD. Boxplots representing the range of contaminant concentrations found in the (**a**) water and (**b**) passive sampler extracts from all samples collected by the citizen scientists across the study. Both the median (solid black line) and mean (dashed black line) for all samples are indicated on the boxplot; the whiskers represent the interquartile range. The coloured dots indicate the location: green = Sheffield, orange = Norwich, and blue = London. Refer to Supplementary Tables 1 to 6 for raw data.

Combined, the citizen science participants collected 50 freshwater samples across the whole study, and a total of 24 unique compounds were detected across all three sampling locations (Sheffield (*n* = 20), Norwich (*n* = 20), and London (*n* = 10)) in this matrix. Of these, 15 compounds were quantifiable (Fig. 1a, refer to Supplementary Tables 1, 2, 3 for LOD/LLOQs) with four compounds (sulfamethoxazole, terbutryn, trimethoprim, and venlafaxine) listed on the European WFD Watch/Priority substances lists^27,28^. Two compounds (tramadol and venlafaxine) were common to at least one sampling site in all three locations. Overall, the average concentration of all contaminants in the water samples from each location was 31 ± 17 ng L^−1^, 40 ± 50 ng L^−1^, and 31 ± 17 ng L^−1^ for Sheffield, Norwich, and London, respectively. On average, the most concentrated compound in the Sheffield, Norwich, and London samples was venlafaxine (40 ± 14 ng L^−1^), propranolol (132 ± 5 ng L^−1^), and bezafibrate (52 ± 27 ng L^−1^), respectively. Of the 25 sites sampled across the whole study, the most frequently occurring compounds in the water samples were venlafaxine (88% of sites), tramadol (56% of sites), citalopram (28% of sites), oxazepam (20% of sites), and ketamine (20% of sites).

The citizen scientists captured a higher spatial resolution compared to sites sampled by the Environment Agency’s (EA) semi-quantitative LC-MS chemical monitoring programme^29^ for the same sampling catchments. Within the Sheffield region, there are no EA monitoring sites (Supplementary Fig. 1a). In the London sampling catchment, there is only one site, but it was not monitored in 2022 (Supplementary Fig. 1b). There are two EA sampling sites in Norwich, but only one was active during the timeframe of this study (Supplementary Fig. 1c). Demonstrating the limited coverage of other large-scale routine sampling campaigns in certain regions and highlights the power of citizen science programmes to help fill these gaps.

**Fig. 2:**
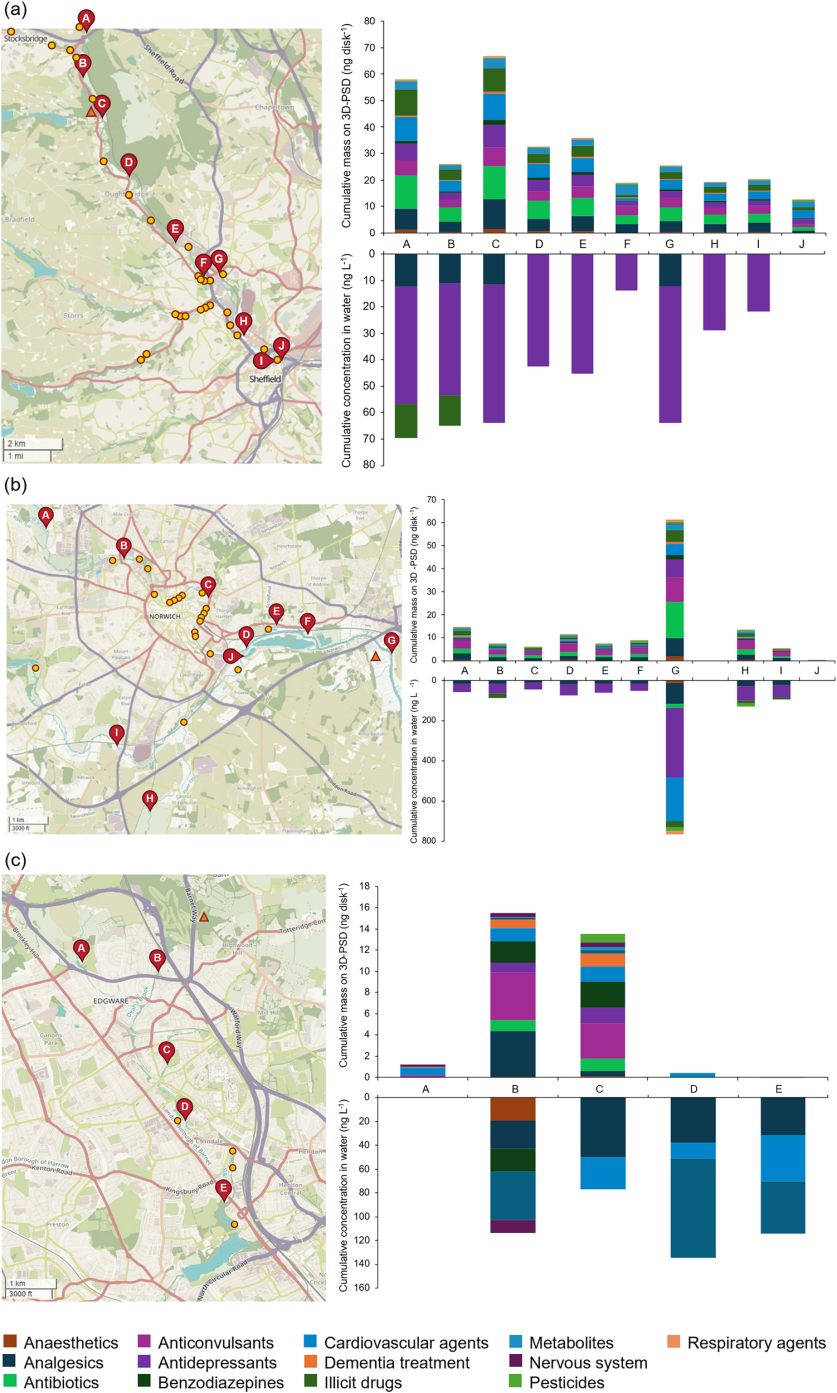
Sampling locations and CECs measured at each site. Map^45^ of the sampling sites along the (**a**) River Don, Sheffield; (**b**) Rivers Yare and Wensum, Norwich; (**c**) Silk Stream, London, as chosen by the community groups (left). Cumulative masses of CECs quantified on the deployed 3D-PSDs by site (top, right), cumulative CEC concentrations quantified in the water samples by site (bottom, right). The numbers represent the total number of compounds quantified at each site per sample type. The locations of active CSOs (yellow circles) and WWTPs (orange triangles) in 2022 are marked^46^. All locations are approximate. Refer to Supplementary Tables 1, 2, 3, 4, 5, and 6 for site GPS coordinates and further compound details. Map data from OpenStreetMap, openstreetmap.org/copyright.

From the water data alone, it was not possible to identify an obvious source of CEC discharge (e.g., combined sewer overflow (CSO), wastewater treatment plant (WWTP) discharge, etc.) along the River Don in Sheffield, as there was no significant increase in the contaminant concentration at any of the sites (Fig. 2 (a), Supplementary Table 1). Contaminants were quantified at all sites except for site Sheff-J where no CECs were present at concentrations above LLOQ, though venlafaxine was present below LLOQ. Across the remaining sites, venlafaxine was present in all samples and accounted for 83% of the total CEC concentrations measured in Sheffield (mean concentration = 40 ± 14 ng L^−1^).

In the Norwich water samples, the CEC pollution impact point was identified geographically close to a treated WWTP discharge outlet (Fig. 2b). Upstream of this location, along both the rivers Yare and Wensum, the average contaminant concentration in water was 25 ± 12 ng L^−1^ for six compounds (acetamiprid, citalopram, ketamine, methamphetamine, tramadol, and venlafaxine), while at site Nor-G, the average concentration in the water was 72 ± 81 ng L^−1^ for 11 compounds (acetamiprid, amitriptyline, bezafibrate, citalopram, diphenhydramine, ketamine, lidocaine, propranolol, tramadol, trimethoprim, and venlafaxine). Six of these (amitriptyline, bezafibrate, diphenhydramine, lidocaine, propranolol, and trimethoprim) were not present at any other sampling site. The presence of these compounds in the Nor-G sample is consistent with compounds found at WWTP outfalls in other studies^5,10,11,30^ (Supplementary Table 2). At site Nor-G, 67% of the combined concentration was represented by venlafaxine (36%), propranolol (17%), and tramadol (14%). Tramadol and venlafaxine were found in nine of the ten sites sampled within the range of 12 to 107 ng L^−1^ and 26 to 280 ng L^−1^, respectively. The highest concentrations for both compounds were observed at site Nor-G. Unfortunately, samples were not taken further downstream of this site, so the dilution of these compounds is unknown. At the two sites located off the main river course, sites Nor-H and Nor-I (Rivers Tas and River Yare, respectively), the average concentrations of contaminants in water was 26 ± 17 ng L^−1^ and 29 ± 17 ng L^−1^, respectively. These concentrations closely align with the concentrations present at the sites upstream of Nor-G, indicating that there are no other significant sources of contamination during the sampling period. At site Nor-J, there were no compounds present at concentrations above the LLOQ for this method. In comparison to the EA semi-quantitative LC-MS chemical monitoring programme dataset, the only active site in 2022 was located between the Nor-A and Nor-B sites and was sampled on the 28^th^ of September 2022, representing the halfway point of the sampling campaign^29^. Of the compounds shared between the EA dataset and our method (*n* = 19), only tramadol and venlafaxine were quantified in both datasets at all three sites. The concentration of venlafaxine is consistent across the sites (EA = 32 ng L^−1^, Nor-A = 30 ± 6 ng L^−1^, and Nor-B = 35 ± 3 ng L^−1^). In comparison, the measured concentration of tramadol (45 ng L^−1^) at the EA site is approximately threefold greater than the concentrations observed in this work (Nor-A = 17 ± 2 ng L^−1^, and Nor-B = 18 ± 1 ng L^−1^). The remaining compounds in the EA dataset were present below the method’s LLOQ used in this study.

In London, four small freshwater systems were captured by the citizen scientists sampling campaign (Burnt Oak Brook, Deans Brook, Edgeware Brook, and the Silk Stream). Site Lon-A was located along the Edgeware Brook and did not have any compounds present in the water samples above the LLOQ of this method (Fig. 2c). The average concentration of contaminants at site Lon-B (Deans Brook) was 23 ± 11 ng L^−1^ and was the only site that contained the herbicide terbutryn. Both systems join into the Silk Stream approximately 2 – 2.5 km downstream from the sampling points. The Lon-C sample was collected from the Burnt Oak Brook, a tributary of the main flow of the Silk Stream, which contained venlafaxine (50 ± 13 ng L^−1^) and lidocaine (27 ± 2 ng L^−1^). Samples from sites Lon-D and Lon-E were collected from the lower regions of the Silk Stream, before it enters the Brent Reservoir. The highest average contaminant concentrations were observed at site Lon-D (45 ± 36 ng L^−1^), driven by the high concentration of bezafibrate (83 ± 7 ng L^−1^, Supplementary Table 3). Bezafibrate is a known marker of CSO discharge^10^ and is also present, though at lower concentrations, at sites Lon-B, Lon-C (below LLOQ), and Lon-E over the range of 33 to 40 ng L^−1^. The source of bezafibrate at Lon-B could be attributed to a CSO (approximate location: 51.633243, -0.250493) that discharges into Deans Brook, located approximately 1.6 km upstream of the sampling site^31^. The closest CSO to site Lon-D is located ~ 400 m upstream, which could justify the higher contaminant concentrations observed at this site^31^.

### Occurrence of contaminants in 3D-PSD extracts

The citizen scientists deployed 25 3D-PSDs across the three sampling locations. Fifty unique compounds were found in the passive sampler extracts, with 25 compounds common across all three locations. There were 33 (Sheffield), 27 (Norwich), and 16 (London) compounds present in the 3D-PSD extracts that were not detectable in the corresponding water sample. The only compound that was quantifiable in the water samples and not in any of the 3D-PSD extracts was methamphetamine, which was only present in the water sampled collected from the Nor-B site on the 30th of September during passive sampler retrieval (Supplementary Table 2). Possibly indicating an acute pollution event. The average concentration of all contaminants on disk for each location was 0.9 ± 1.0 ng disk^−1^, 0.6 ± 1.0 ng disk^−1^, and 0.5 ± 0.8 ng disk^−1^ for Sheffield, Norwich, and London, respectively.

In Sheffield, 39 unique CECs across all sampling sites were quantified using the 3D-PSD. There were 30 compounds present at all sites, with the most concentrated being tramadol (opioid pain killer), carbamazepine (anticonvulsant), and ketamine (illicit drug), representing 12, 12, and 11% of the total CEC concentrations measured in Sheffield, respectively. Twelve compounds were present in both the Sheffield samples and blanks, of which eight (amiodarone, ketoprofen, levocabastine, methylphenidate, salicylic acid, spinosyn A, sulfamethoxazole, and verapamil) were removed from the analysis of all sites due to contamination. The remaining four compounds were included in the analysis for at least one sampling site (refer to Supplementary Table 4 for details). The Sheffield 3D-PSD data revealed two possible pollution sources into the River Don from the relatively high cumulative concentrations of CECs accumulated on the sampling disk at these locations (Shef-A and Shef-C) compared to the other sites (Fig. 2a). At both locations, the average mass on disk across all CECs at these two locations was 1.6 ± 2.0 ng disk^−1^ (Shef-A) and 1.9 ± 2.0 ng disk^−1^ (Shef-C) in comparison to the other sampling locations, where the average mass on disk ranged from 0.4 ± 0.5 ng disk^−1^ to 1.0 ± 1.0 ng disk^−1^. This suggests that a discharge event from CSOs located near or upstream of the Shef-A and Shef-C sites during the deployment period was not captured in the water samples collected by the citizen scientists. When the results of these two sites were queried with the citizen scientists during the final results presentation, they confirmed that there were WWTPs within 5 km of both the Shef-A and Shef-C sampling sites.

At the Norwich sites, 42 unique CECs were quantified in the 3D-PSD extracts. Of these, carbamazepine and salbutamol (asthma medication) were common to all sites monitored using the 3D-PSD. Eighteen compounds were present in both the sample and blank extracts, of which five (amiodarone, cocaine, cymoxanil, flufenoxuron, and levocabastine) were completely removed from the analysis across all sites due to contamination. The remaining 13 compounds were included in the analysis for at least one sampling site (refer to Supplementary Table 5 for details). The 3D-PSD data from the Norwich sampling sites followed a similar trend to the water chemicals data, indicating a pollution source near site Nor-G. The average mass of contaminants on disk for site Nor-G was 1.5 ± 2 ng disk^-1^ for 44 compounds, representing an almost five-fold increase in contaminant mass compared to the upstream site along the River Yare and indicating its proximity to an active WWTP discharge point (Fig. 2b). The average concentration of contaminants on the 3D-PSD at these other sites was < 0.5 ng disk^-1^, indicating no other significant sources of contamination along the monitored stretches of the River Yare during this sampling period. The citizen scientists also placed two 3D-PSDs in two tributaries that feed into the River Yare, the River Tas (Nor-H) and the River Wensum (Nor-I). The participants also chose to deploy one sampler in the little lake behind the kayaker’s clubhouse (Nor-J, Fig. 2b). The average concentration of compounds at the Nor-H and Nor-I sites (0.5 ± 0.8 ng disk^-1^ and 0.3 ± 0.5 ng disk^-1^, respectively) indicated no significant sources of contamination from the two tributaries. The lowest contaminant concentrations were observed in the extracts from the Nor-J site (0.05 ± 0.04 ng disk^-1^), likely due to dilution and the very low flow observed in the little lake, which affects contaminant uptake onto the passive sampler^32–34^.

Across all the sites selected by the citizen scientists in the London community group, 35 unique CECs were quantified in the 3D-PSD extracts. Due to high levels of contamination in the field blanks, no compound was common to all five London sites, but bisoprolol (a beta-blocker) and imidacloprid (a neonicotinoid pesticide) were all present in 80% of the sites. Five compounds (antipyrine, citalopram, levocabastine, methylphenidate, and verapamil) were completely removed from the analysis across all sites due to contamination. Overall, the average concentration of contaminants on the London 3D-PSDs was lower than that observed in the Sheffield and Norwich sampling campaigns. The highest average concentration on the 3D-PSD was found to be at site Lon-B, while the highest average concentration of contaminants was observed at site Lon-D from the water data (Fig. 2c). Unfortunately, the corresponding field blank from the Lon-D site was highly contaminated, and as a result, almost all of the compounds present in that sample were removed from the analysis (refer to Supplementary Table 6 for details). The 3D-PSD at Lon-E had very few contaminants accumulated on disk (bisoprolol = 0.01 ng disk^-1^), potentially due to the lack of flow at the deployment location, or the 3D-PSD was not fully submerged throughout the sampling campaign.

### Environmental risk assessment

**Fig. 3:**
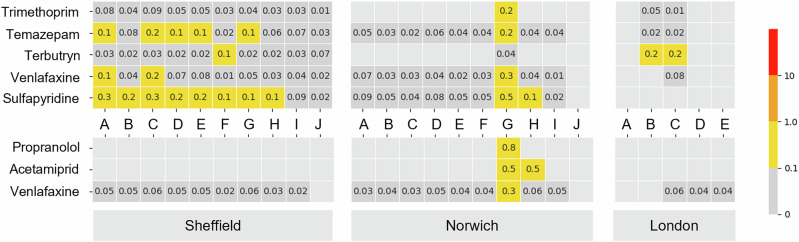
Environmental risk assessment per sampling location. Heatmap of the highest risk quotients (*RQs*) calculated using water measurements (bottom) and the TWA concentrations derived from the 3D-PSDs for all sites (top). Light grey cells indicate non-detects, and the calculated *RQ* values are annotated to one significant figure. Refer to Supplementary Figure 2 for full *RQ* data and PNEC values used. Refer to Supplementary Figure 3 for the values used to calculate the *RQs*.

Across all the study sites, *RQ*s could be calculated for 29 compounds (Fig. 3). Due to the lack of *Rs* values, *RQs* could not be calculated for 58% (*n* = 29) of the 50 compounds quantified in the 3D-PSD extracts. Of these, imidacloprid (neonicotinoid insecticide) had the lowest PNEC of 6.8 ng L^-1^ ^35^. For the compounds for which the *RQs* could be calculated, 22 were calculated to have an *RQ* < 0.1 (negligible risk) across all sampling sites and methods (refer to Supplementary Fig. 2 for full details). The remaining seven (acetamiprid, propranolol, sulfapyridine, temazepam, terbutryn, trimethoprim, and venlafaxine) were calculated to have an *RQ* > 0.1 (low risk, Fig. 3). No compounds were calculated to have medium or high risk at any sites. For compounds where the *RQs* could be calculated in both the water and 3D-PSD extracts, there was generally good agreement between the categories of risk (i.e., negligible and low), though the *RQ* values tended to be slightly higher in the 3D-PSD samples compared to the water data for the same sites. There were two instances when the *RQ* classification differed between the 3D-PSD and the water data. Both trimethoprim and venlafaxine were classified as having a low risk from the 3D-PSD data and a negligible risk from the water data (Fig. 3). This is due to the higher TWA concentration in water calculated for these compounds from the uptake onto the 3D-PSD than those directly measured in the water sample (Supplementary Fig. 3). It is difficult to compare the calculated TWA concentrations to infrequent spot sampling, as pollution events may have been missed depending on the time the water sample was collected. Sulfapyridine, temazepam, and terbutryn were not present in water samples above LLOQ, and the *RQs* for the 3D-PSDs for acetamiprid and propranolol could not be calculated due to the lack of *Rs* values.

### Participants’ experience and insights

To assess the value of blue-green spaces to active river users, the participants were surveyed before the commencement of the study (refer to Supplementary Note 1 and Supplementary Note 3 for the pre- and post-study surveys and Supplementary Tables 7, 8, 9, and Supplementary Notes 2 and 4 for the results). On average, participants spent four to five days a week at/on the river or visited it at least once per month. The time participants spent on/at the river varied from 30 min to 4 h, depending on their activity. Over half the participants reported attending the river alone, though visits with others (family, friends, partners, community groups, etc.) were common and, in those instances, the group size varied between three to over ten individuals.

When asked to describe their local rivers in a single word, the participants provided a range of responses, which could be grouped into positive, negative or factual/observational, depending on the context. Most of the positive comments were associated with leisure activities such *as ‘kayaking’* and *‘paddling’* as well as positive emotions such as *‘relaxing’*, *‘fun’*, and *‘freedom’*. The negative comments mainly pertained to sensory language (i.e., sight and smell) used to describe their river environment, such as *‘brown’*, *‘smell/smelly’*, and *‘dirty’*. The participants were also asked which river contaminants were of most interest/concern to them and provided a wide range of responses from general categories such as *‘sewage’*, *‘poisons’*, and *‘chemicals/chemical waste’* to more specific contaminants such as *‘oil’*, *‘iron and mercury’*, and *‘artificial hormones’*. The participants were also interested in *‘bacteria’, ‘microbes’*, *‘microplastics’*, and *‘viruses’*, indicating that the participants had an enhanced awareness of potential river pollutants and hazards to public health.

The study results were presented to the citizen scientists via Zoom in November 2022, and a report was privately disseminated to the participants a year later in November 2023. The meeting allowed the participants to discuss results with the research team and attendees from other organisations, brainstorm ideas for future projects, and collect feedback. Overall, the feedback from the participants regarding the results delivery format was positive, with many participants actively interested in the chemical composition of the different rivers, how the pollution dilutes as it moves downstream, and the concentrations of contaminants that were found in the rivers from human sources. One participant made the poignant comment, *“I’d never really thought about how much we interact with and engage with the water system around us as a family and individuals.”* Another stated: *“Don’t take our rivers for granted, it looks clean but there’s so much more to do; there’s more work to be done to change people’s attitudes to chemicals and disposal.”* These statements provided insight as to how the experience with the study prompted the participants to reflect on the value of their local rivers and the impact of chemical pollution. Critically, when polled, 90% of the participants said that they would be interested in taking part in a study like this again.

The citizen scientists also provided feedback on the experience of deploying and retrieving the 3D-PSDs and collecting the water samples. They identified a range of challenges with the protocol that was adapted from one designed and followed by research scientists^23^. These included difficulties when putting gloves on with wet hands, complicated instructions, and the challenges of recording site details when the record sheet was wet. The participants also experienced some of the typical challenges of field work (such as sample deployment taking longer than expected, finding suitable locations for the 3D-PSDs, and safely navigating the river’s currents). This feedback is vitally important for improving deployment methods and protocols, and for making them more user-friendly for future participatory research studies. Despite the challenges, the participants also noted positive experiences, including having fun, finding it interesting to assess different deployment locations, and enjoying the experience of traveling to sites via canoe/kayak to explore new river areas.

## Discussion

This work represents the first time scalable 3D-PSDs have been used by citizen scientists to monitor CECs across multiple freshwater catchments. Seventeen citizen scientists from three different community groups were recruited and successfully trained to independently deploy and retrieve 25 3D-PSDs and collect 50 water samples at locations of their choice along waterways in Sheffield, Norwich, and London. A total of 51 different CECs were quantified in the water and 3D-PSD extracts.

Across all sampling sites, 24 contaminants were detected in the water samples, of which 15 were quantifiable. In comparison, 50 contaminants were present in the 3D-PSD extracts, including compounds that were not detectable in the corresponding water samples. Demonstrating the increased selectivity of passive sampling compared to the direct analysis of water grab samples due to its *in-situ* accumulation properties. However, acute pollution events can be missed by the passive sampler due to the rate of compound diffusion from the environment to the sorbent. The use of a preconcentration step, such as solid-phase extraction (SPE), could have increased the number of contaminants detected/quantified in the water samples. However, the direct analysis of river water samples does not suffer from the imprecision and a more limited chemical space selectivity that often arises when using advanced and active sample preparation techniques, such as SPE^7^. The method used in this study is also high-throughput, which is ideal for large-scale monitoring campaigns, while maintaining good and environmentally relevant sensitivity limits (LOD = 3 ± 5 ng L^-1^, LLOQ = 9 ± 17 ng L^-1^), and represents a more sustainable analysis approach due to the reduced solvent consumption compared to SPE^30^.

Sites with CEC discharge from pollution sources were readily identifiable in water and 3D-PSD extracts from the significant increase in the total concentration of contaminants compared to other sites, and the presence of compounds known to be present in discharge from CSOs and WWTPs (e.g., amitriptyline, bezafibrate, diphenhydramine, lidocaine, propranolol, and trimethoprim)^5,10,11,30^. Across all sampling sites, compounds were removed from the passive sampler analysis due to their presence in the transport and field blanks at levels exceeding the designated threshold. The source of contamination is unclear, though it could be due to the inexperience of the citizen scientists with scientific fieldwork or handling the blanks.

The environmental risk quotients (*RQ*) for compounds were assessed, and the concentrations present were all below the medium-risk threshold with relatively good agreement between the water and 3D-PSD data. However, the *RQs* could not be assessed for 29 compounds present in the 3D-PSD extracts due to the lack of uptake rate values. Of these, imidacloprid had the lowest PNEC of 6.8 ng L^-1^ ^35^. Imidacloprid is a neonicotinoid insecticide that has been withdrawn from outdoor agricultural use in the EU and UK since 2018 (transcribed into UK law following its withdrawal from the European Union) due to concerns over its effects on non-target invertebrates, such as bees, though it also has considerable veterinary applications as a flea and tick treatment for domestic pets^36–40^. Despite the bans, multiple studies post-2020 have reported the presence of imidacloprid in urban freshwater systems at concentrations posing an environmental risk^5,7,23,39,41,42^. In this work, imidacloprid was present in the 3D-PSD extracts from 92% of sites across the whole study. Given the prevalence and very high risk posed by this compound, further work is needed to determine the passive sampler uptake rates for this compound in order to better assess its environmental risk.

The study participants highlighted the importance of blue river spaces in the daily lives of urban residents and their potential to impact their fitness and mental health, as well as contribute to other community-building and social interactions. A limitation of this work is that our study population is biased towards urban river users, and non-river or rural river users may have a different perspective. The participants also demonstrated an awareness of potential river pollutants and their impact on public health. This study specifically focused on chemical contaminants; future work focusing on the detection of harmful viruses and bacteria was clearly of interest to these participants. Feedback from the study participants indicated that they enjoyed participating in the study and gained new knowledge and understanding of chemical pollution in their local rivers. However, they did identify areas for improvement for future work, including streamlining the deployment procedure and improving the data entry/collection system.

This study demonstrated the feasibility of using the 3D-PSD as an alternative to traditional grab sampling campaigns for more practical spatiotemporal coverage in future large-scale community engagement studies. This work also demonstrated the benefits to both the participants and scientists from engaging in citizen science projects, where both parties gained new knowledge and understanding.

## Methods

### Reagents and consumables

All reagents used were at least HPLC-MS grade or higher unless stated otherwise. Organic solvents including methanol (MeOH), acetonitrile (MeCN), and propan-2-ol (IPA) were obtained from VWR Scientific (Leicestershire, UK). Formic acid was purchased from Millipore (Millipore, Bedford, USA). Ultra-pure water was dispensed from a Millipore Milli-Q water purification system (MilliporeSigma, Massachusetts, USA) at 18.2 MΩ.cm. For quantitative targeted analysis, a standard mix of 200 CEC compounds with a purity of ≥ 97% was used, including 36 stable isotope-labelled internal standards (SIL-IS). Target compounds include pharmaceuticals and personal care products (PPCPs, *n* = 95), pesticides (*n* = 56), illicit substances (*n* = 10), and human metabolites (*n* = 3). These compounds were selected for a variety of reasons, including global occurrence in freshwater systems, human consumption/usage (e.g., prescription, agricultural use, etc.), known or suspected to cause ecological harm, and listed in UK and EU regulations^43^. Refer to Supplementary Note 5 for full details.

### 3D-PSD fabrication and preparation for deployment

A total of 25 3D-printed passive samplers were manufactured using an Asiga Max (Puretone^TM^ Ltd., Kent, UK) using the commercially available methacrylate-based resin (PlasCLEAR v2). The full design, development, and characterisation of the 3D-PSD is described in Richardson et al*.* (2022), including details on its manufacture, assembly, calibration, and downloadable *.STL files^23^. The 3D-PSD is a planar passive sampler similar in design to the Chemcatcher® device but the new design allows for five 9 mm sorbents to be contained within the same device. The 3D-PSD housing consists of two core components, a top and a base, and a removable transport cap. All pieces fit and hold together through a friction interference fit (Supplementary Fig. 4a). Printed components underwent two 15 min washes in IPA using a sonicator to remove excess resin from the manufacturing process. Once dry, the parts were cured for 30 min under UV light using the Asiga Flash UV oven (Puretone^TM^ Ltd., Kent, UK). All parts were rinsed with MeOH and water before assembly.

The 3D-PSD housings were loaded with five hydrophilic–lipophilic balanced (HLB) sorbent discs (9 mm diameter) consisting of a divinylbenzene polymer-based sorbent backbone functionalized with hydrophilic moieties and 0.2 µm Supor poly(ether sulphone) (PES) membranes punched to the same size (9 mm diameter) using an appropriately sized leather wad punch. The PES membranes were used to protect the HLB sorbent from biofouling and act as a diffusion limiter. The HLB sorbents were acquired from Affinisep (AttractSPE® Discs HLB, Val de Reuil, France), and the PES membrane was purchased from Pall Europe Ltd. (Portsmouth, UK). Excess manufacturing residues from the PES membrane were removed with duplicate 24-hour washes of MeOH before assembly and deployment. The HLB sorbent disk was conditioned by soaking sequentially in MeOH, followed by ultrapure water for 24 h. The 3D-PSDs were assembled by first placing the PES membrane inside the upside-down lid component and then placing the HLB sorbent disk on top before fitting the base component to the lid and pressing the device together. Refer to Richardson et al. (2022) for further details^23^. Assembled devices were stored in ultrapure water for at least 48 h before deployment. All 3D-PSDs were fitted with a small cable tie with a unique identification number.

### Participant recruitment

This study was conducted with ethical approval from Imperial College Science, Engineering and Technology Research Ethics Committee (SETREC, reference:22IC7769). River users were specifically targeted as participants for this work as it was highly likely that they would have a vested interest in their local rivers, be familiar with possible pollution sources, be comfortable in the water, and be aware of the potential hazards that working in a river presents.

A total of 17 citizen scientists from four different community groups were recruited using the following process. A variety of community groups were identified online (e.g., watersports clubs, angler clubs/societies) and contacted with a brief overview of the project and an informational flyer (Supplementary Note 6). Interested individuals or representatives of a community were invited to an online interview, carried out in July/August of 2022, to further explain project details and determine their group’s suitability. This was also an opportunity for the citizen scientists to ask questions about the project and better understand the goals and motivation of the study. Following this call, the community group leaders recruited at least five participants from within their own community and arranged an in-person training date with the scientific team.

### Procedure for the citizen scientists to deploy the 3D-PSDs in their local rivers

Prior to all deployments, all citizen scientists participated in a three-hour in-person training session led by the scientific team near their local river. During this session, the citizen scientists learned different 3D-PSD deployment methods (including the controls necessary for scientific integrity), were trained to collect water samples, and were provided with all the necessary equipment. At the end of the session, the citizen scientists were provided with all the equipment and 3D-PSDs needed to complete the planned deployments. Informed consent to participate in the study was obtained from all participants before the training session.

In total, 25 deployment sites were selected by the citizen scientists along freshwater rivers in three major cities (Sheffield, Norwich, and London). Refer to Supplementary Tables 1, 2, 3 for GPS coordinates of deployment locations. Ten 3D-PSDs were deployed by the Sheffield (River Don) and Norwich (Rivers Yare, Wensum and Tas) participants, while five were deployed along the Silk Stream and associated tributaries (Burnt Oak Brook, Deans Brook, and Edgeware Brook) by the community group in a London area. All deployment locations were chosen by the participants based on their local river knowledge, with advice given by the scientific team regarding site selection (e.g., sampling at a range of both suspected polluted and non-polluted sites, safe site access for the participants, and considering accessibility by the general public to minimise device tampering/removal). Sampling for all community groups occurred over the same period from the 24th of September 2022 to the 2nd of October 2022.

Working in pairs, the citizen scientists travelled to each location either on foot or via canoe/kayak. Once at the chosen location, the citizen scientists deployed the 3D-PSD up-to one metre under the surface of the water using the most appropriate method depending on the location. These include a) fitting the 3D-PSD onto a U-shaped garden peg (15 × 0.29 cm, G&B, UK) and pushing it into the riverbed so that the passive sampler was face up and 5–10 cm above the surface of the river bed (Supplementary Fig. 4b); b) cable tying the 3D-PSD to a knot on a rope which was threaded through a ~ 300 g weight and anchored to a small buoy or the river bank (Supplementary Fig. 4c); c) cable tying the 3D-PSD to an existing fixed structure (e.g., underside of a pier, submerged steel rod, etc.) where it consistently remained below the water level (Supplementary Fig. 4d). Citizen scientists were encouraged to be creative with deployment methods, with the main criterion being that the 3D-PSDs were always below the water level. At each location, citizen scientists exposed a field blank (an assembled 3D-PSD exposed to the ambient environment) during deployment and retrieval to account for any contamination that may have occurred during handling. The participants filled out a datasheet detailing the unique five-digit ID number of the deployed passive sampler and the field blank used, the time and date, the deployment location (using www.what3words.com) and any additional comments (e.g., signs of pollution, land use, river flow, weather, etc.). Water samples for each site were collected during the 3D-PSD deployment and one week later at retrieval using 30 mL pre-rinsed Nalgene® bottles (Sigma-Aldrich, UK). Participants were instructed to rinse the Nalgene® bottle three times with river water before collecting the final sample and to record the time and dates the water samples were collected.

After seven days, the citizen scientists retrieved the 3D-PSDs from the river and vigorously shook the device underwater to dislodge any particulates/debris. The samplers were wrapped in dry MeOH-washed aluminium foil that was a part of the sampling equipment provided (Supplementary Note 7) and placed into a plastic bag in a cooler box with icepacks. Within each community group, one participant triaged all the 3D-PSDs and water samples from the group for collection by overnight courier to the laboratory for analysis.

### 3D-PSD and water extraction procedures

Once received in the laboratory, the 3D-PSDs were first rinsed in ultrapure water, and then the HLB sorbent discs and PES membrane were removed from the housing. The HLB sorbents were left to dry overnight on fresh MEOH-washed aluminium foil alongside the corresponding field blank. Once dry, the sorbent discs were stored at -20 °C until sample preparation. Compound uptake on the PES membrane after 7 days was briefly assessed (refer to Supplementary Table 10 for results) during the initial uptake experiments as detailed in Richardson et al. (2022)^23^. Briefly, five litres of artificial freshwater was spiked to 50 ng L^-1^ with a mix of 164 analytical standards and agitated using a magnetic stirrer. Fully assembled 3D-PSDs (HLB sorbents and PES membranes) were exposed within the beaker for seven days, with the fortified AFW replaced daily. An unused, fully assembled 3D-PSD was exposed during set-up and sample collection to act as a negative control. The HLB sorbents and PES membranes were prepared and analysed as described in the methods. A calibration curve was prepared from spare PES membranes exposed in the same experimental set-up over the range of 0.005 to 1 ng. To reduce the impact of matrix effects arising from biofilms present on the PES membrane, it was discarded and did not undergo any further analysis.

Extraction of the 9 mm HLB sorbent discs followed the process described by Richardson et al. (2022)^23^, with a small adjustment to reduce the incidence of LC-system blockage during analysis. In brief, dry HLB discs (*n* = 3 HLB discs per 3D-PSD) were spiked to 0.5 ng disk^-1^ with 36 SIL-IS in MeOH and extracted in an empty, fritted SPE cartridge (polypropylene, 20 μm pores, Agilent Technologies UK Ltd., Cheshire, UK) configured to a standard SPE manifold. With the taps closed, 1 mL of MeOH was added to the cartridge and left to soak for 15 min. After this time, the tap was opened, and the extraction solvent was allowed to fall into 2 mL microcentrifuge tubes (Eppendorf, Stevenage, UK). An additional 0.6 mL of MeOH was added on top and gently pulled through under vacuum. Extracts were passed through a 0.2 µm polytetrafluoroethylene (PTFE) filter membrane using a BD Plastipak^TM^ syringe (FisherScientific UK Ltd., Loughborough, UK) into 15 mL evaporation tubes. Extracts were fully evaporated at 35 °C under a gentle flow of N_2_ and reconstituted in 200 µL of LC-MS starting mobile phase. Quantification was performed by conditioning fresh HLB sorbent discs in MeOH and allowing them to dry before spiking with analytical standards to form an eleven-point calibration series (0.005 to 10 ng disk^-1^) and extracted as above. During data processing, for compounds where the signal in any of the blanks (field, laboratory, or transport) was greater than 10% of the sample signal, the compound was removed from the analysis as the possibility of contamination could not be ruled out^23^. Passive sampler extracts were stored at -20 °C until analysis.

Water samples were prepared for analysis as per the methods described in Egli et al. (2021)^30^ and Richardson et al. (2021)^7^. In summary, 900 µL of river water was spiked with 100 µL of standards and SIL-IS to the required concentration (500 ng L^-1^ for the SIL-IS). Quantification was performed using an external matrix-matched calibration curve (5–2000 ng L^-1^, *n* = 12) prepared from a pooled sample containing equal volumes of water from each corresponding sampling location from the different community groups. Samples were briefly vortexed before filtering through 0.2 µm syringe filter directly into deactivated HPLC vials (Agilent A-Line, Agilent Technologies UK Ltd., Didcot, UK). Water samples were stored at -20 °C until analysis.

### Instrumental analysis

All 3D-PSD extracts and grab water samples were analysed using a targeted LC-MS/MS method. Separations were performed on a Restek Raptor 5.0 × 3.0 mm, 2.7 µm biphenyl guard column (Restek, Pennsylvania, USA) using a validated method on a LCMS-8060 instrument (Shimadzu Corporation, Kyoto, Japan)^23,30,44^. The elution gradient comprised of an initial hold of 90:10 mobile phase A (MPA, 0.1% *v/v* formic acid (aq)):mobile phase B (MPB, 0.1% *v/v* formic acid in 50:50 MeOH:MeCN) for 0.2 min, followed by a linear ramp to 60% MPB over 2.8 min, an 100% MPB hold for 1 min, before returning to initial conditions for a 1.5 min re-equilibration time. All samples were analysed with an injection volume of 10 µL using a 0.5 mL min^-1^ flow rate. All data was acquired and processed using Shimadzu LabSolutions and LabSolutions Insights LCMS, respectively.

### Environmental risk assessment

*RQ* were calculated as the ratio (Eqn. 1) between the concentration measured in the environment and the predicted no-effect concentration (PNEC) values sourced from the NORMAN Ecotoxicological Database^35^. The environmental concentrations were measured either directly from the water samples or from the time-weighted average (TWA) contaminant concentration in water determined from the mass accumulated on the 3D-PSD and a known uptake rate constant (*R*_*s*_). Refer to Richardson et al. for the *R*_*s*_ values for the HLB-orientated 3D-PSDs^23^. To align with previous research, the risk thresholds were assigned as: insignificant risk ( < 0.1), low risk (0.1–1), medium risk (1–10), and high risk ( > 10)^5,30^.

1 $${RQ}=\frac{{MEC}}{{PNEC}}$$

## Supplementary information

Supplementary Information

## Data Availability

Measurement data from the EA Water Quality Monitoring programme was downloaded from the Department for Environment Food & Rural Affairs (DEFRA) Data Services Platform, https://environment.data.gov.uk/water-quality/view/download and https://www.data.gov.uk/dataset/0c63b33e-0e34-45bb-a779-16a8c3a4b3f7/water-quality-monitoring-data-gc-ms-and-lc-ms-semi-quantitative-screen. Summarised data generated during this study are presented in the supplementary file. The raw data is available from the corresponding author upon reasonable request.
