# Supplementary material for: A community-guided approach to monitoring contaminants of emerging concern in freshwater systems using passive samplers
Source: NPJ Emerg Contam. 2026 Jan 7;2(1):1. doi: 10.1038/s44454-025-00021-1 (PMC12863633; doi:10.1038/s44454-025-00021-1)
Supplement: Supplementary file 1 — Supplementary Information [file 44454_2025_21_MOESM1_ESM.pdf]

Supplementary Material

A community-guided approach to monitoring contaminants of emerging concern in freshwater systems using passive samplers

Alexandra K Richardson[1*], Stav Friedman[1], and Leon P Barron[1]

[1] *MRC Centre for Environment & Health, Environmental Research Group, School of Public Health, Faculty of Medicine, Imperial College London, 86 Wood Lane, London W12 0BZ, United Kingdom.*

* Corresponding author: a.richardson@imperial.ac.uk

Supplementary Table 1. Mean and standard deviations of contaminants (ng L^{-1}) detected in each of the water samples collected by the Sheffield citizen scientists along with LOD and LLOQs [1]. Compounds present below the LLOQ are indicated.

Compound	LOD (ng L^{-1})	LLOQ (ng L^{-1})	A		B		C		D		E		F		G		H		I		J	
			53.483708, -1.559579		53.472119, -1.56216		53.455625, -1.551336		53.440855, -1.539017		53.421127, -1.516508		53.412341, -1.493463		53.41032, -1.499434		53.393852, -1.482062		53.385471, -1.464328		53.385309, -1.462156	
			25-Sep	02-Oct	25-Sep	02-Oct	25-Sep	02-Oct	25-Sep	02-Oct	25-Sep	02-Oct	25-Sep	02-Oct	25-Sep	02-Oct	25-Sep	02-Oct	25-Sep	02-Oct	25-Sep	02-Oct
Benzoylecgonine	4	11	D	D	D	D	D	D	D	D	D	D	D	D	D	D	D	D	D	D	D	D
Carbamazepine	4	12	D	D	D	D	D	D	D	D	D	D	D	D	D	D	D	D	D	D	D	D
Citalopram	4	13	-	< LLOQ	< LLOQ	< LLOQ	< LLOQ	< LLOQ	< LLOQ	< LLOQ	-	< LLOQ	-	-	-	< LLOQ	-	-	-	-	-	-
Clarithromycin	5	16	D	D	D	D	D	D	-	-	D	D	-	-	D	-	-	-	-	D	-	-
Diclofenac	4	13	D	D	D	D	D	D	D	D	D	D	D	D	D	D	D	D	D	D	D	D
Ketamine	4	11	< LLOQ	13 ± 0.51	< LLOQ	11 ± 0.17	< LLOQ	< LLOQ	< LLOQ	< LLOQ	< LLOQ	< LLOQ	-	-	< LLOQ	< LLOQ	-	< LLOQ	-	-	-	-
Lidocaine	4	11	-	< LLOQ	-	-	-	< LLOQ	-	-	-	< LLOQ	-	-	-	< LLOQ	-	-	-	-	-	-
Nortriptyline	4	11	-	-	-	-	-	-	-	< LLOQ	-	-	-	-	-	-	< LLOQ	-	-	-	-	-
Propanolol	6	19	D	D	D	D	D	D	D	D	D	D	D	D	D	D	D	D	D	D	D	D
Tramadol	4	11	< LLOQ	12 ± 0.3	< LLOQ	11 ± 0.06	12 ± 0.27	12 ± 0.37	< LLOQ	< LLOQ	< LLOQ	< LLOQ	< LLOQ	< LLOQ	12 ± 0.15	12 ± 0.41	< LLOQ	< LLOQ	< LLOQ	-	-	-
Venlafaxine	4	11	35 ± 3.91	55 ± 5.08	30 ± 3.16	55 ± 2.48	44 ± 0.38	60 ± 3.17	35 ± 1.34	50 ± 2.31	37	54	< LLOQ	14 ± 5.41	45 ± 1.32	58 ± 2.17	22 ± 0.59	36 ± 3.51	19 ± 0	24 ± 0	< LLOQ	< LLOQ

D: compound was not able to be quantified (calibration was not fit for purpose (R2 < 0.98)), but a clear chromatographic peak is visible; -: not detected

Supplementary Table 2. Mean and standard deviations of contaminants (ng L^{-1}) detected in each of the water samples collected by the Norwich citizen scientists along with LOD and LLOQs [1].. Compounds present below the LLOQ are indicated.

Compound	LOD (ng L^{-1})	LLOQ (ng L^{-1})	A		B		C		D		E		F		G		H		I		J	
			52.647437, 1.248068		52.641508, 1.278585		52.631805, 1.308258		52.620701, 1.323006		52.624043, 1.345127		52.626065, 1.335577		52.618518, 1.376488		52.584048, 1.287202		52.59856, 1.2754		52.61949, 1.32258	
			23-Sep	30-Sep	23-Sep	30-Sep	23-Sep	30-Sep	23-Sep	30-Sep	24-Sep	01-Oct	24-Sep	01-Oct	24-Sep	01-Oct	24-Sep	30-Sep	24-Sep	30-Sep	24-Sep	01-Oct
Acetamiprid	4	11	< LLOQ	-	< LLOQ	< LLOQ	< LLOQ	-	< LLOQ	-	< LLOQ	< LLOQ	< LLOQ	< LLOQ	13 ± 3	26 ± 3	19 ± 3	< LLOQ	< LLOQ	< LLOQ	< LLOQ	-
Amitriptyline	4	11	-	-	-	-	-	-	-	-	-	-	-	-	< LLOQ	13 ± 4	-	-	-	-	-	-
Atrazine	3	10	D	D	D	D	D	D	D	D	D	D	D	D	D	D	D	D	D	D	D	D
Benzoylecgonine	4	11	D	D	D	D	D	D	D	D	D	D	D	D	D	D	D	D	D	D	D	D
Bezafibrate	8	25	-	-	-	-	-	-	-	-	-	-	-	-	< LLOQ	83 ± 19	-	-	-	-	-	-
Bisoprolol	4	11	D	D	D	D	D	D	D	D	D	D	D	D	D	D	D	D	D	D	D	D
Carbamazepine	4	12	D	D	D	D	D	D	D	D	D	D	D	D	D	D	D	D	D	D	D	D
Carbamazepine-10, 11-epoxy	4	11	-	-	-	-	-	-	-	-	-	-	-	-	< LLOQ	< LLOQ	-	-	-	-	-	-
Citalopram	4	13	14 ± 3	< LLOQ	< LLOQ	14 ± 2	< LLOQ	< LLOQ	< LLOQ	14 ± 3	14 ± 2	< LLOQ	< LLOQ	< LLOQ	54 ± 5	62 ± 1	14 ± 1	18 ± 1	20 ± 2	15 ± 2	< LLOQ	< LLOQ
Clarithromycin	5	16	-	D	-	-	-	-	-	-	-	-	-	-	D	D	-	D	D	-	-	-
Clopidogrel	4	11	D	D	D	D	D	D	D	D	D	D	D	D	D	D	D	D	D	D	D	D
Clozapine	4	11	-	-	-	-	-	-	-	-	-	-	-	-	-	< LLOQ	-	-	-	-	-	-
Cocaine	4	11	D	D	D	D	D	D	D	D	D	D	D	D	D	D	D	D	D	D	D	D
Diclofenac	4	13	D	D	D	D	D	D	D	D	D	D	D	D	D	D	D	D	D	D	D	D
Diphenhydramine	4	11	-	-	-	-	-	-	-	-	-	-	-	-	15 ± 0.2	20 ± 3	-	-	-	-	-	-
Disulfoton sulfone	0	0	D	D	D	D	D	D	D	D	D	D	D	D	D	D	D	D	D	D	D	D
Fluoxetine	4	11	D	D	-	D	D	D	D	-	D	D	D	D	D	D	D	D	D	D	D	D
Ketamine	4	11	< LLOQ	< LLOQ	< LLOQ	< LLOQ	< LLOQ	< LLOQ	< LLOQ	< LLOQ	< LLOQ	< LLOQ	< LLOQ	< LLOQ	30 ± 1	28 ± 0.9	< LLOQ	13 ± 0.7	13 ± 1	-	-	-
Lidocaine	4	11	-	-	-	-	-	-	-	-	-	-	-	-	12 ± 0.6	12 ± 0.5	-	-	-	-	-	-
Memantine	4	13	-	-	-	-	-	-	-	-	-	-	-	-	< LLOQ	< LLOQ	-	-	-	-	-	-
Methamphetamine	4	11	-	-	-	22 ± 1	-	-	-	-	-	-	-	-	-	-	-	-	-	-	-	-
Methylphenidate	4	11	D	D	D	D	D	D	D	D	D	D	D	D	D	D	D	D	D	D	D	D
Propanolol	6	19	-	-	-	< LLOQ	-	-	-	-	< LLOQ	-	-	-	127 ± 8	137 ± 17	-	< LLOQ	< LLOQ	-	-	-
Risperidone	3	10	D	D	D	D	D	D	D	D	D	D	D	D	D	D	D	D	D	D	D	D
Salbutamol	4	11	D	D	D	D	D	D	D	D	D	D	D	D	D	D	D	D	D	D	D	D
Sulfapyridine	4	11	-	-	-	-	-	-	-	-	-	-	-	-	< LLOQ	< LLOQ	-	-	-	-	-	-
Temazepam	4	11	D	D	D	D	D	D	D	D	D	D	D	D	D	D	D	D	D	D	D	D
Tramadol	4	11	19 ± 0.8	14 ± 1	18 ± 0.3	17 ± 1	14 ± 0.4	14 ± 0.6	20 ± 1	21 ± 0.2	18 ± 0.9	14 ± 1	19 ± 0.6	17 ± 1	107 ± 2	106 ± 4	29 ± 1	32 ± 0.3	36 ± 1	12 ± 0.9	-	-
Trimethoprim	3	10	-	-	-	-	-	-	-	-	-	-	-	-	17 ± 1	21 ± 2	-	-	-	-	-	-
Venlafaxine	4	11	35 ± 6	26 ± 3	36 ± 4	34 ± 3	31 ± 8	30 ± 3	44 ± 3	39 ± 4	34 ± 2	31 ± 2	37 ± 2	32 ± 8	270 ± 4	282 ± 11	50 ± 4	58 ± 2	58 ± 3	27 ± 6	-	-

D: compound was not able to be quantified (calibration was not fit for purpose (R2 < 0.98)), but a clear chromatographic peak is visible; -: not detected

Supplementary Table 3. Mean and standard deviations of contaminants (ng L^{-1}) detected in each of the water samples collected by the London citizen scientists along with LOD and LLOQs [1].. Compounds present below the LLOQ are indicated.

Compound	LOD (ng L-^{1})	LLOQ (ng L^{-1})	A		B		C		D		E	
			51.623693, -0.284484		51.62282, -0.26392		51.606282, -0.260916		51.595825, -0.256836		51.580247, -0.244732	
			25-Sep	02-Oct	25-Sep	02-Oct	25-Sep	02-Oct	25-Sep	02-Oct	25-Sep	02-Oct
Atrazine	3	10	D	D	D	D	D	D	D	D	D	D
Benzoylecgonine	4	11	-	-	< LLOQ	< LLOQ	-	-	< LLOQ	< LLOQ	< LLOQ	< LLOQ
Bezafibrate	8	25	-	-	< LLOQ	40 ± 4	< LLOQ	< LLOQ	83 ± 7	-	33 ± 2	< LLOQ
Bisoprolol	4	11	D	D	D	D	D	D	D	D	D	D
Carbamazepine	4	12	D	D	D	D	D	D	D	D	D	D
Cocaine	4	11	-	-	-	-	< LLOQ	< LLOQ	< LLOQ	< LLOQ	< LLOQ	< LLOQ
Diclofenac	4	13	D	D	D	D	D	D	D	D	D	D
Diphenhydramine	4	11	D	D	D	D	D	D	D	D	D	D
Disulfoton sulfone	0	0	D	D	D	D	D	D	D	D	D	D
Haloperidol	4	13	D	D	D	D	D	D	D	D	D	D
Ketamine	4	11	D	D	D	D	D	D	D	D	D	D
Lidocaine	4	11	-	< LLOQ	< LLOQ	< LLOQ	< LLOQ	27 ± 2	13 ± 3	< LLOQ	48 ± 1	29 ± 0.6
Methamphetamine	4	11	-	-	-	< LLOQ	-	-	< LLOQ	-	-	-
Oxycodone	4	11	D	D	D	D	D	D	D	D	D	D
Prometryn	4	12	D	D	D	D	D	D	D	D	D	D
Salbutamol	3	10	D	D	D	D	D	D	D	D	D	D
Sulfamethoxazole	4	11	-	-	25 ± 0.8	14 ± 0.3	-	-	-	-	< LLOQ	-
Sulfapyridine	4	11	-	-	-	-	-	-	< LLOQ	-	< LLOQ	< LLOQ
Terbutryn	4	11	-	-	< LLOQ	11 ± 1.3	< LLOQ	< LLOQ	-	< LLOQ	-	< LLOQ
Tramadol	4	11	< LLOQ	< LLOQ	19 ± 0.9	< LLOQ	-	< LLOQ	< LLOQ	< LLOQ	< LLOQ	< LLOQ
Trimethoprim	4	11	D	D	D	D	D	D	D	D	D	D
Valsartan	3	10	-	-	-	-	-	-	< LLOQ	< LLOQ	11 ± 0.9	11 ± 0.6
Venlafaxine	3	10	-	-	16 ± 3	31 ± 4	38 ± 4	62 ± 3	38 ± 4	38 ± 7	32 ± 1	30 ± 0.2

D: compound was not able to be quantified (calibration was not fit for purpose (R2 < 0.98)), but a clear chromatographic peak is visible; -: not detected

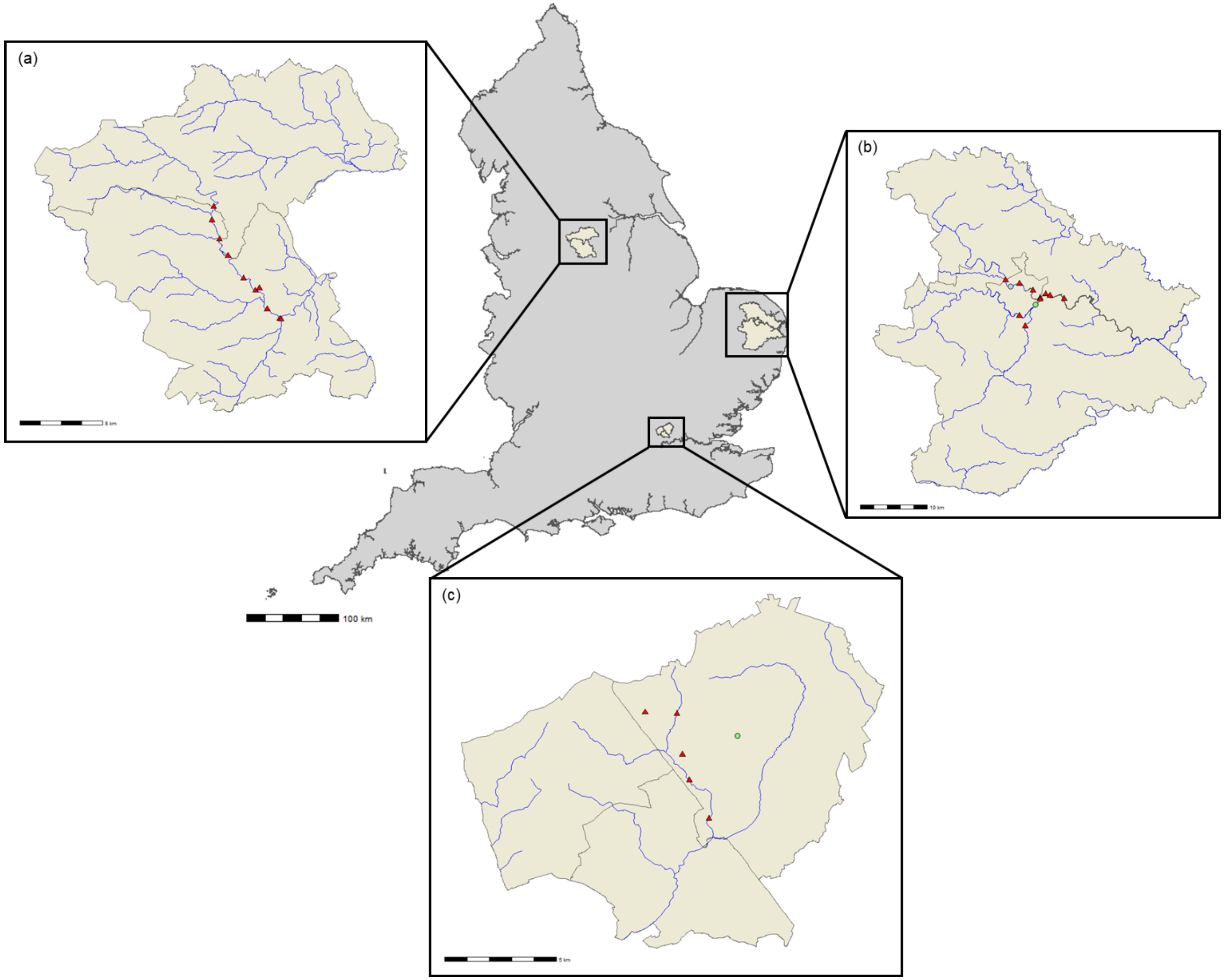

Supplementary Figure 1. *Map of sampling sites as chosen by the citizen scientists (red) in comparison to sites monitored by the Environment Agency[2] in the sampling catchment area (green = all time, blue = 2022).*

Supplementary Table 4. Mean and standard deviations of contaminants (ng $disk^{-1}$) detected using the 3D-PSDs deployed by the Sheffield citizen scientists along with LODs and LLOQs [1]. Compounds present below the LLOQ are indicated

Compound	LOD (ng $disk^{-1}$)	LLOQ (ng $disk^{-1}$)	A	B	C	D	E	F	G	H	I	J
			53.483708, -1.559579	53.472119, -1.56216	53.455625, -1.551336	53.440855, -1.539017	53.421127, -1.516508	53.412341, -1.493463	53.41032, -1.499434	53.393852, -1.482062	53.385471, -1.464328	53.385309, -1.462156
Acetamiprid	0.002	0.007	0.02 ± 0.01	0.01 ± 0.003	0.03 ± 0.003	0.02 ± 0.002	0.01 ± 0.011	< LLOQ	0.01 ± 0.002	0.01 ± 0.003	< LLOQ	< LLOQ
Amitriptyline	0.04	0.1	0.79 ± 0.2	0.32 ± 0.07	1.02 ± 0.2	0.57 ± 0.2	0.56 ± 0.1	0.21 ± 0.06	0.26 ± 0.06	0.24 ± 0.07	0.28 ± 0.03	0.15 ± 0.01
Atorvastatin	0.07	0.2	D	D	D	-	-	-	-	-	-	-
Atrazine	0.04	0.1	B	B	B	B	B	B	B	B	B	0.38 ± 0.03
Azithromycin	0.02	0.05	D	D	D	D	D	B	D	D	B	B
Benzoylecgonine	0.008	0.02	1.77 ± 0.1	1.01 ± 0.02	1.78 ± 0.04	1.14 ± 0.1	1.17 ± 0.03	3.23 ± 0.3	1.03 ± 0.03	0.86 ± 0.06	0.93 ± 0.08	1.89 ± 0.2
Bezafibrate	0.02	0.05	0.55 ± 0.04	0.26 ± 0.04	0.63 ± 0.02	0.34 ± 0.05	0.38 ± 0.04	0.07 ± 0.03	0.27 ± 0.02	0.19 ± 0.03	0.2 ± 0.02	0.08 ± 0.02
Bisoprolol	0.002	0.005	1.6 ± 0.2	0.74 ± 0.02	2 ± 0.2	0.83 ± 0.1	0.83 ± 0.02	0.2 ± 0.02	0.61 ± 0.01	0.44 ± 0.04	0.45 ± 0.03	0.16 ± 0.02
Carbamazepine	0.006	0.02	5.95 ± 0.5	3.02 ± 0.1	7.31 ± 0.5	3.68 ± 0.6	4.3 ± 0.3	3.51 ± 0.5	3.88 ± 0.05	2.71 ± 0.1	2.81 ± 0.3	1.2 ± 0.1
Carbamazepine-10, 11-epoxy	0.002	0.006	1.2 ± 0.09	0.64 ± 0.04	2.06 ± 0.2	1 ± 0.2	1.15 ± 0.07	0.6 ± 0.07	0.89 ± 0.03	0.6 ± 0.03	0.65 ± 0.04	0.21 ± 0.03
Citalopram	0.03	0.1	2.22 ± 0.2	0.96 ± 0.2	3.06 ± 0.3	1.65 ± 0.4	1.71 ± 0.1	0.53 ± 0.08	0.9 ± 0.2	0.77 ± 0.2	0.83 ± 0.05	0.66 ± 0.09
Clarithromycin	0.04	0.1	4.31 ± 0.5	1.69 ± 0.1	5.25 ± 0.7	2.22 ± 0.6	2.38 ± 0.4	0.4 ± 0.1	1.25 ± 0.2	1.09 ± 0.4	1.19 ± 0.3	0.58 ± 0.1
Clopidogrel	0.007	0.02	0.39 ± 0.05	0.17 ± 0.02	0.47 ± 0.02	0.17 ± 0.04	0.2 ± 0.03	0.08 ± 0.02	0.15 ± 0.2	0.09 ± 0.01	0.12 ± 0.02	0.12 ± 0.02
Cocaine	0.01	0.04	0.43 ± 0.05	0.24 ± 0.008	0.42 ± 0.04	0.19 ± 0.03	0.19 ± 0.01	0.81 ± 0.1	0.13 ± 0.005	0.14 ± 0.02	0.16 ± 0.02	0.56 ± 0.07
Diclofenac	0.1	0.3	0.96 ± 0.09	0.39 ± 0.07	0.9 ± 0.08	< LLOQ	0.72 ± 0.6	0.59 ± 0.8	0.37 ± 0.04	< LLOQ	< LLOQ	< LLOQ
Diphenhydramine	0.01	0.04	0.43 ± 0.05	0.13 ± 0.006	0.44 ± 0.06	0.25 ± 0.09	0.24 ± 0.03	< LLOQ	0.1 ± 0.02	0.06 ± 0.03	0.07 ± 0.01	< LLOQ
Fluoxetine	0.04	0.1	0.58 ± 0.2	B	0.64 ± 0.08	0.38 ± 0.1	0.34 ± 0.09	B	B	B	B	B
Imidacloprid	0.02	0.05	0.66 ± 0.05	0.36 ± 0.06	0.85 ± 0.06	0.51 ± 0.05	0.54 ± 0.04	0.21 ± 0.04	0.38 ± 0.05	0.28 ± 0.04	0.29 ± 0.07	0.23 ± 0.07
Ketamine	0.006	0.02	9.24 ± 2.9	3.64 ± 0.5	7.89 ± 2	3.33 ± 0.7	3.86 ± 0.7	0.09 ± 0.03	2.59 ± 0.6	2.12 ± 0.3	2.01 ± 0.2	0.38 ± 0.06
Levamisole	0.01	0.04	0.13 ± 0.03	0.08 ± 0.03	0.2 ± 0.08	0.1 ± 0.03	0.12 ± 0.04	0.09 ± 0.03	0.1 ± 0.02	0.07 ± 0.02	0.05 ± 0.02	< LLOQ
Lidocaine	0.001	0.003	1.06 ± 0.2	0.47 ± 0.05	1.31 ± 0.2	0.57 ± 0.1	0.64 ± 0.08	0.17 ± 0.04	0.46 ± 0.05	0.37 ± 0.04	0.32 ± 0.02	0.14 ± 0.02
MDMA	0.008	0.03	0.31 ± 0.03	0.13 ± 0.02	0.36 ± 0.03	0.14 ± 0.04	0.13 ± 0.03	0.07 ± 0.02	0.08 ± 0.04	0.07 ± 0.02	0.09 ± 0.01	0.09 ± 0.02
Memantine	0.005	0.01	0.59 ± 0.1	0.2 ± 0.03	0.83 ± 0.1	0.32 ± 0.1	0.37 ± 0.1	0.05 ± 0.02	0.18 ± 0.04	0.16 ± 0.04	0.21 ± 0.02	0.03 ± 0.008

Supplementary Table 4 cont.

Metoprolol	0.003	0.01	0.1 ± 0.007	0.05 ± 0.003	0.11 ± 0.01	0.05 ± 0.007	0.05 ± 0.008	< LLOQ	0.04 ± 0.002	0.03 ± 0.002	0.03 ± 0.003	< LLOQ
Nicotine	0.01	0.04	D	D	D	B	D	D	B	D	D	D
Nordiazepam	0.01	0.04	0.13 ± 0.03	0.07 ± 0.02	0.19 ± 0.04	0.11 ± 0.03	0.12 ± 0.02	0.04 ± 0.007	0.08 ± 0.03	0.06 ± 0.02	0.08 ± 0.03	0.04 ± 0.01
Nortriptyline	0.03	0.1	0.51 ± 0.1	0.26 ± 0.07	0.56 ± 0.03	0.38 ± 0.1	0.41 ± 0.09	0.18 ± 0.1	0.23 ± 0.05	0.23 ± 0.07	0.25 ± 0.03	0.16 ± 0.04
Oxazepam	0.006	0.02	0.43 ± 0.07	0.24 ± 0.03	0.65 ± 0.05	0.33 ± 0.07	0.37 ± 0.06	0.13 ± 0.03	0.31 ± 0.06	0.26 ± 0.05	0.29 ± 0.04	0.14 ± 0.05
Prometryn	0.01	0.03	-	-	-	-	-	0.04 ± 0.01	-	-	-	-
Propranolol	0.01	0.04	2.96 ± 0.3	1.27 ± 0.2	3.02 ± 0.2	1.66 ± 0.3	1.71 ± 0.1	0.58 ± 0.1	1.03 ± 0.1	0.88 ± 0.2	0.97 ± 0.05	0.39 ± 0.03
Salbutamol	0.01	0.04	0.11 ± 0.005	0.05 ± 0.02	0.19 ± 0.01	0.1 ± 0.009	0.1 ± 0.003	0.06 ± 0.007	0.08 ± 0.004	0.06 ± 0.02	0.06 ± 0.01	0.04 ± 0.02
Sertraline	0.05	0.2	0.96 ± 0.3	0.39 ± 0.1	1.15 ± 0.08	0.7 ± 0.3	0.61 ± 0.1	0.47 ± 0.2	0.32 ± 0.06	0.36 ± 0.2	0.3 ± 0.04	0.68 ± 0.1
Sulfamethazine	0.02	0.07	< LLOQ	< LLOQ	< LLOQ	< LLOQ	< LLOQ	0.07 ± 0.01	< LLOQ	< LLOQ	< LLOQ	< LLOQ
Sulfapyridine	0.02	0.06	6.46 ± 0.6	3.16 ± 0.05	5.3 ± 0.4	3.26 ± 0.4	3.34 ± 0.1	2.16 ± 0.2	2.93 ± 0.09	1.91 ± 0.1	1.69 ± 0.2	0.48 ± 0.06
Tamsulosin	0.005	0.02	0.09 ± 0.01	0.05 ± 0.006	0.11 ± 0.009	0.06 ± 0.009	0.06 ± 0.009	< LLOQ	0.05 ± 0.006	0.03 ± 0.008	0.04 ± 0.004	0.02 ± 0.007
Temazepam	0.004	0.01	0.6 ± 0.06	0.32 ± 0.03	0.91 ± 0.06	0.39 ± 0.08	0.47 ± 0.05	0.1 ± 0.02	0.39 ± 0.03	0.26 ± 0.03	0.29 ± 0.04	0.14 ± 0.008
Terbutryn	0.004	0.01	0.09 ± 0.009	0.05 ± 0.008	0.08 ± 0.009	0.06 ± 0.009	0.07 ± 0.01	0.4 ± 0.1	0.05 ± 0.006	0.06 ± 0.007	0.08 ± 0.01	0.19 ± 0.03
Timolol	0.001	0.002	0.06 ± 0.007	0.04 ± 0.006	0.07 ± 0.007	0.03 ± 0.007	0.04 ± 0.008	0.01 ± 0.004	0.03 ± 0.005	0.03 ± 0.003	0.03 ± 0.008	0.02 ± 0.005
Tramadol	0.003	0.009	6.47 ± 0.6	2.88 ± 0.2	9.46 ± 0.8	4.29 ± 1	4.58 ± 0.2	2.39 ± 0.7	3.32 ± 0.4	2.76 ± 0.5	3.2 ± 0.09	0.51 ± 0.07
Trimethoprim	0.004	0.01	1.67 ± 0.2	0.77 ± 0.02	2.02 ± 0.1	1.21 ± 0.2	1.12 ± 0.04	0.76 ± 0.1	0.89 ± 0.07	0.61 ± 0.03	0.59 ± 0.04	0.2 ± 0.03
Valsartan	0.04	0.1	3.15 ± 0.2	1.44 ± 0.1	3.51 ± 0.1	1.83 ± 0.3	2.09 ± 0.2	0.35 ± 0.1	1.44 ± 0.09	0.97 ± 0.2	1.14 ± 0.1	2.25 ± 0.3
Venlafaxine	0.008	0.03	1.06 ± 0.1	0.4 ± 0.01	2.01 ± 0.2	0.69 ± 0.2	0.77 ± 0.01	0.09 ± 0.01	0.46 ± 0.03	0.32 ± 0.05	0.36 ± 0.02	0.24 ± 0.03

D: compound was not able to be quantified (calibration was not fit for purpose (R2 < 0.98)), but a clear chromatographic peak is visible; B: signal in field blanks > 10% of that in the sample; -: not detected

Supplementary Table 5. Mean and standard deviations of contaminants (ng disk^{-1}) detected using the 3D-PSDs deployed by the Norwich citizen scientists along with LODs and LLOQs[1]. Compounds present below the LLOQ are indicated

Compound	LOD (ng disk^{-1})	LLOQ (ng disk^{-1})	A	B	C	D	E	F	G	H	I	J
			52.647437, 1.248068	52.641508, 1.278585	52.631805, 1.308258	52.620701, 1.323006	52.624043, 1.345127	52.626065, 1.335577	52.618518, 1.376488	52.584048, 1.287202	52.59856, 1.2754	52.61949, 1.32258
Acetamiprid	0.002	0.007	< LLOQ	0.01 ± 0.003	0.02 ± 0.003	0.01 ± 0.002	-	0.02 ± 0.003	0.02 ± 0.009	0.01 ± 0.002	-	-
Amitriptyline	0.04	0.1	-	-	-	-	-	-	0.52 ± 0.2	-	-	-
Atrazine	0.04	0.1	0.36 ± 0.04	0.14 ± 0.03	0.18 ± 0.02	0.3 ± 0.01	0.18 ± 0.02	0.24 ± 0.05	0.56 ± 0.1	0.73 ± 0.3	B	B
Azithromycin	0.02	0.05	-	-	-	-	-	-	D	-	-	-
Azoxystrobin	0.009	0.03	< LLOQ	-	-	0.05 ± 0.003	0.06 ± 0.008	0.08 ± 0.006	0.07 ± 0.008	-	-	-
Benzoylecgonine	0.008	0.02	0.28 ± 0.02	0.18 ± 0.02	B	0.27 ± 0.009	B	B	B	0.3 ± 0.02	B	B
Bezafibrate	0.02	0.05	0.1 ± 0.008	0.05 ± 0.01	< LLOQ	0.14 ± 0.006	0.09 ± 0.007	0.09 ± 0.005	0.87 ± 0.07	0.2 ± 0.02	< LLOQ	-
Bisoprolol	0.002	0.005	0.06 ± 0.007	0.03 ± 0.005	0.03 ± 0.004	0.04 ± 0.004	0.03 ± 0.008	0.04 ± 0.004	0.95 ± 0.05	0.07 ± 0.01	0.02 ± 0.003	-
Carbamazepine	0.006	0.02	3.49 ± 0.3	1.97 ± 0.2	1.78 ± 0.1	3.82 ± 0.1	2.49 ± 0.1	2.82 ± 0.09	10.43 ± 0.4	3.61 ± 0.1	2.26 ± 0.1	0.05 ± 0.008
Carbamazepine-10, 11-epoxy	0.002	0.006	0.6 ± 0.05	0.3 ± 0.03	0.26 ± 0.02	0.66 ± 0.03	0.4 ± 0.01	0.43 ± 0.04	2.56 ± 0.1	0.58 ± 0.02	0.29 ± 0.02	-
Citalopram	0.03	0.1	B	B	B	B	B	B	4.19 ± 0.9	B	B	B
Clarithromycin	0.04	0.1	0.16 ± 0.01	0.13 ± 0.03	0.15 ± 0.03	0.12 ± 0.01	< LLOQ	0.2 ± 0.03	1.72 ± 0.6	0.24 ± 0.06	< LLOQ	-
Clopidogrel	0.007	0.02	0.04 ± 0.006	-	< LLOQ	0.02 ± 0.005	0.02 ± 0.007	0.02 ± 0.006	0.65 ± 0.05	0.03 ± 0.009	-	-
Clothianidin	0.05	0.1	0.28 ± 0.05	0.15 ± 0.04	-	< LLOQ	0.17 ± 0.02	0.16 ± 0.03	-	-	-	-
Clozapine	0.02	0.06	-	-	-	-	-	-	D	-	-	-
Diclofenac	0.1	0.3	< LLOQ	< LLOQ	< LLOQ	< LLOQ	< LLOQ	< LLOQ	1.57 ± 0.2	0.4 ± 0.07	< LLOQ	-
Diphenhydramine	0.01	0.04	< LLOQ	-	-	-	< LLOQ	-	0.44 ± 0.2	< LLOQ	-	-
Fluoxetine	0.04	0.1	-	B	B	-	-	B	0.35 ± 0.06	B	-	B
Hydrochlorothiazide	0.09	0.3	< LLOQ	-	-	-	-	-	0.32 ± 0.05	< LLOQ	-	-
Imidacloprid	0.02	0.05	0.47 ± 0.06	0.28 ± 0.05	0.25 ± 0.07	0.57 ± 0.04	0.37 ± 0.06	0.32 ± 0.04	1.35 ± 0.1	0.44 ± 0.04	0.37 ± 0.03	B
Ketamine	0.006	0.02	1.98 ± 0.2	0.76 ± 0.3	0.63 ± 0.1	0.86 ± 0.05	0.63 ± 0.06	0.68 ± 0.08	4.42 ± 2	1.37 ± 0.7	0.38 ± 0.05	-
Lidocaine	0.001	0.003	0.43 ± 0.05	0.18 ± 0.07	0.17 ± 0.02	0.23 ± 0.02	0.16 ± 0.02	0.21 ± 0.06	1.68 ± 0.5	0.33 ± 0.1	0.17 ± 0.01	-
MDMA	0.008	0.03	-	-	-	-	-	-	0.6 ± 0.2	-	-	-
Memantine	0.005	0.01	0.43 ± 0.08	0.2 ± 0.05	0.14 ± 0.02	0.2 ± 0.008	0.16 ± 0.02	0.15 ± 0.01	0.78 ± 0.3	0.14 ± 0.05	0.09 ± 0.01	-
Methylphenidate	0.005	0.01	B	B	B	B	B	B	0.16 ± 0.07	B	B	B
Metoprolol	0.003	0.01	0.02 ± 0.007	0.01 ± 0.002	0.01 ± 0.005	0.01 ± 0.004	0.01 ± 0.005	0.01 ± 0.003	0.12 ± 0.01	0.01 ± 0.003	< LLOQ	-
Nicotine	0.01	0.04	B	B	B	B	B	B	D	B	B	B
Nordiazepam	0.01	0.03	0.07 ± 0.02	< LLOQ	< LLOQ	0.06 ± 0.01	0.04 ± 0.01	0.05 ± 0.02	0.31 ± 0.04	0.04 ± 0.01	0.05 ± 0.009	-
Nortriptyline	0.03	0.1	-	< LLOQ	< LLOQ	< LLOQ	< LLOQ	< LLOQ	0.41 ± 0.03	< LLOQ	< LLOQ	-
Oxazepam	0.006	0.01	0.17 ± 0.02	B	B	0.2 ± 0.04	0.14 ± 0.02	0.18 ± 0.03	0.78 ± 0.04	0.15 ± 0.02	B	B
Pirenzipine	0.01	0.03	-	-	-	-	-	-	0.03 ± 0.005	-	-	-
Propanolol	0.01	0.04	0.28 ± 0.05	0.16 ± 0.03	0.18 ± 0.03	0.25 ± 0.02	0.16 ± 0.04	0.18 ± 0.02	2.97 ± 0.3	0.36 ± 0.03	0.07 ± 0.02	-

Supplementary Table 5. cont.

Salbutamol	0.01	0.038	0.1 ± 0.003	0.06 ± 0.02	0.05 ± 0.01	0.09 ± 0.009	0.06 ± 0.01	0.07 ± 0.02	0.34 ± 0.01	0.1 ± 0.007	0.04 ± 0.008	0.05 ± 0.005
Simazine	0.2	0.5	D	D	D	D	D	D	D	-	D	-
Sulfamethoxazole	0.01	0.03	B	B	B	B	B	B	1.2 ± 0.06	B	B	B
Sulfapyridine	0.02	0.06	1.81 ± 0.1	1.06 ± 0.07	0.75 ± 0.06	1.54 ± 0.07	0.95 ± 0.04	1.02 ± 0.07	8.93 ± 0.4	2.13 ± 0.2	0.41 ± 0.02	-
Tamsulosin	0.005	0.01	-	-	-	-	-	-	0.11 ± 0.01	0.02 ± 0.006	-	-
Temazepam	0.004	0.01	0.19 ± 0.02	0.11 ± 0.01	0.09 ± 0.009	0.24 ± 0.02	0.15 ± 0.02	0.17 ± 0.03	0.89 ± 0.04	0.15 ± 0.02	0.16 ± 0.01	-
Terbutryn	0.004	0.01	B	B	B	B	B	B	0.11 ± 0.006	B	B	B
Thiacloprid	0.004	0.01	-	-	-	-	-	-	0.07 ± 0.01	-	-	-
Timolol	0.001	0.002	0.04 ± 0.008	0.02 ± 0.002	0.02 ± 0.003	0.03 ± 0.009	0.02 ± 0.006	0.02 ± 0.005	0.08 ± 0.005	0.03 ± 0.005	-	-
Tramadol	0.003	0.009	2.33 ± 0.2	1.01 ± 0.2	0.92 ± 0.1	1.28 ± 0.09	0.83 ± 0.2	1.08 ± 0.3	5.32 ± 2	1.55 ± 0.6	0.8 ± 0.08	B
Trimethoprim	0.004	0.01	B	B	B	B	B	B	3.88 ± 0.3	B	B	B
Valsartan	0.04	0.1	0.25 ± 0.05	0.18 ± 0.03	B	0.24 ± 0.03	0.14 ± 0.03	0.21 ± 0.02	0.22 ± 0.01	0.3 ± 0.03	-	-
Venlafaxine	0.008	0.02	0.68 ± 0.08	0.29 ± 0.01	0.26 ± 0.02	0.35 ± 0.02	0.21 ± 0.05	0.32 ± 0.08	3.12 ± 0.6	0.37 ± 0.08	0.14 ± 0.01	-

D: compound was not able to be quantified (calibration was not fit for purpose (R2 < 0.98)), but a clear chromatographic peak; B: signal in field blanks > 10% of that in the sample;-: not detected

Supplementary Table 6. Mean and standard deviations of contaminants (ng disk^{-1}) detected using the 3D-PSDs deployed by the London citizen scientists along with LODs and LLOQs [1]. Compounds present below the LLOQ are indicated.

Compound	LOD (ng disk^{-1})	LLOQ (ng disk^{-1})	A	B	C	D	E
			51.623693, -0.284484	51.62282, -0.26392	51.606282, -0.260916	51.595825, -0.256836	51.580247, -0.244732
Acetamiprid	0.002	0.007	< LLOQ	< LLOQ	< LLOQ	B	< LLOQ
Ametryn	0.008	0.02	0.03 ± 0.006	-	-	-	-
Atrazine	0.04	0.1	< LLOQ	0.26 ± 0.02	0.47 ± 0.01	B	-
Azithromycin	0.02	0.05	-	-	-	D	-
Azoxystrobin	0.009	0.03	-	-	< LLOQ	B	-
Benzoylecgonine	0.008	0.02	0.07 ± 0.03	4.41 ± 0.1	2.88 ± 0.2	B	B
Bezafibrate	0.02	0.05	-	0.09 ± 0.02	-	B	-
Bisoprolol	0.002	0.005	0.01 ± 0.005	0.15 ± 0.06	0.25 ± 0.05	B	0.01 ± 0.008
Carbamazepine	0.006	0.02	0.06 ± 0.06	0.2 ± 0.09	0.3 ± 0.1	B	-
Carbamazepine-10,11-epoxy	0.002	0.006	0.01 ± 0.006	0.07 ± 0.01	0.4 ± 0.004	B	< LLOQ
Clarithromycin	0.04	0.1	< LLOQ	0.14 ± 0.007	0.27 ± 0.02	B	-
Clopidogrel	0.007	0.02	-	-	0.02 ± 0.02	B	-
Cocaine	0.01	0.04	B	0.51 ± 0.02	0.82 ± 0.02	B	B
Diclofenac	0.1	0.3	-	< LLOQ	0.38 ± 0.05	B	-
Hydrochlorothiazide	0.09	0.3	-	-	< LLOQ	B	-
Imidacloprid	0.02	0.05	0.37 ± 0.009	0.26 ± 0.01	0.39 ± 0.007	0.4 ± 0.03	-
Ketamine	0.006	0.02	0.05 ± 0.004	0.18 ± 0.008	0.29 ± 0.007	B	-
Lidocaine	0.001	0.003	0.19 ± 0.005	0.35 ± 0.007	0.44 ± 0.04	B	-
MDMA	0.008	0.03	0.03 ± 0.005	< LLOQ	0.07 ± 0.01	B	-
Memantine	0.005	0.01	-	-	0.04 ± 0.002	B	-
Metoprolol	0.003	0.01	-	0.08 ± 0.02	0.04 ± 0.02	B	-
Nicotine	0.01	0.04	B	2.07 ± 0.05	2.5 ± 0.1	B	B
Oxazepam	0.006	0.02	-	-	0.18 ± 0.002	B	-
Oxycarboxin	0.007	0.02	B	B	0.11 ± 0.03	B	B
Prometryn	0.01	0.03	-	0.12 ± 0.1	0.1 ± 0.09	B	-
Propanolol	0.01	0.04	0.09 ± 0.008	0.24 ± 0.01	0.14 ± 0.003	B	-
Salbutamol	0.01	0.04	0.05 ± 0.003	0.06 ± 0.01	0.11 ± 0.002	B	< LLOQ
Simazine	0.2	0.5	-	D	D	-	-
Sulfamethoxazole	0.01	0.03	B	3.06 ± 0.3	B	B	B

Supplementary Table 6 cont.

Sulfapyridine	0.02	0.06	< LLOQ	-	< LLOQ	B	-
Temazepam	0.004	0.01	-	0.07 ± 0.003	0.1 ± 0.001	B	-
Terbutryn	0.004	0.01	-	0.69 ± 0.007	0.52 ± 0.02	B	-
Timolol	0.001	0.002	-	0.03 ± 0.09	0.03 ± 0.04	B	-
Tramadol	0.003	0.009	B	0.92 ± 0.08	0.65 ± 0.03	B	B
Trimethoprim	0.004	0.01	-	1.08 ± 0.02	0.24 ± 0.01	B	-
Valsartan	0.04	0.1	-	0.32 ± 0.3	0.99 ± 0.2	B	-
Venlafaxine	0.008	0.03	-	-	0.8 ± 0.01	B	-

D: compound was not able to be quantified (calibration was not fit for purpose (R2 < 0.98)), but a clear chromatographic peak is visible; B: signal in field blanks > 10% of that in the sample; -: not detected

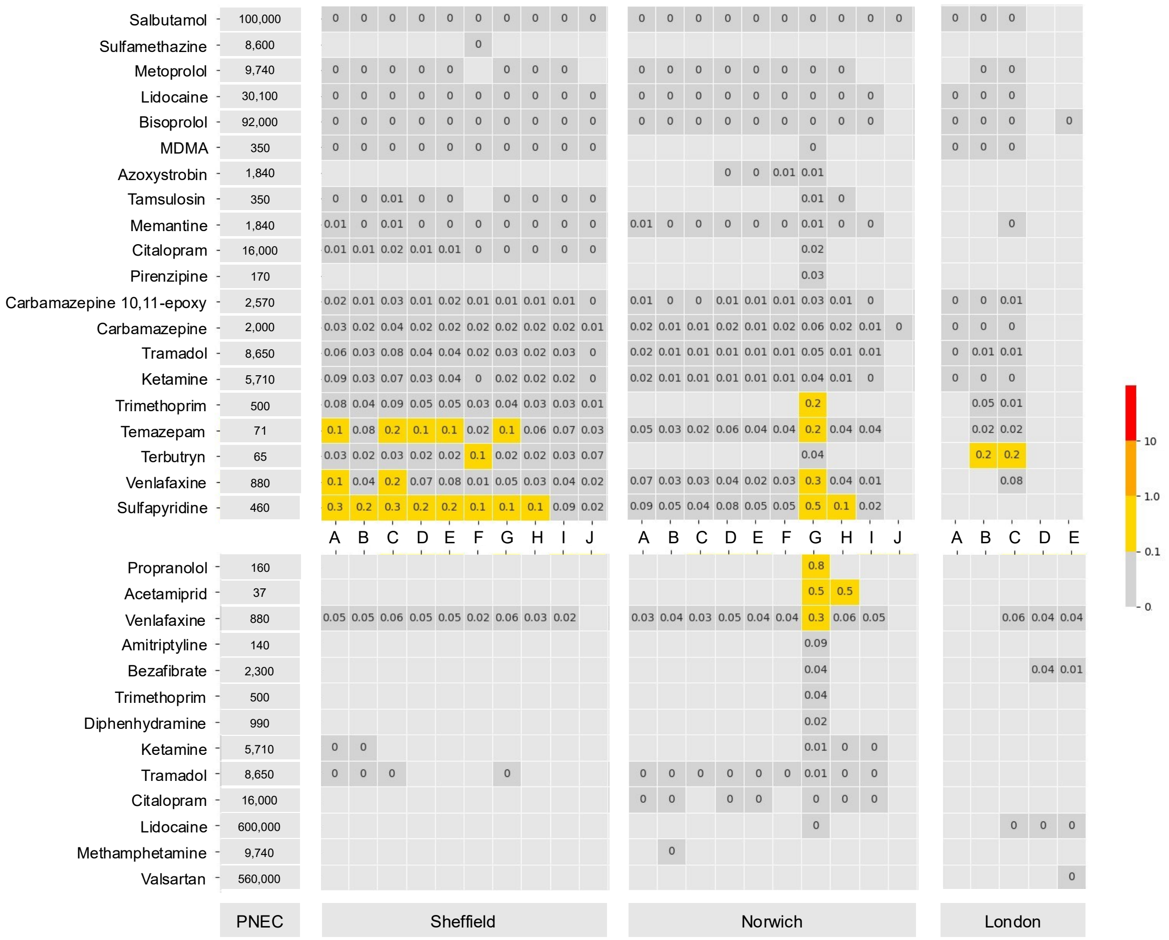

Supplementary Figure 2. *Heatmap of risk quotients (RQs) calculated using water measurements (bottom) and the TWA concentrations derived from the 3D-PSDs for all sites (top). Calculated RQ values are annotated to one significant figure and values <0.01 are represented as 0. Light grey cells indicated not detected and the freshwater PNEC values used are provided (ng L^{-1})* [3].

	Rs	Sheffield										Norwich										London				
		A	B	C	D	E	F	G	H	I	J	A	B	C	D	E	F	G	H	I	J	A	B	C	D	E
Salbutamol	4.8	3	2	6	3	3	2	2	2	2	1	3	2	2	3	2	2	10	3	1	2	1	2	3		
Sulfamethazine	6.0						2																			
Metoprolol	4.1	4	2	4	2	2		1	1	1		0.8	0.4	0.4	0.4	0.4	0.4	4	0.4				3	1		
Lidocaine	0.8	189	84	233	102	115	30	83	66	58	25	77	32	31	42	29	38	300	58	31		35	63	78		
Bisoprolol	3.7	62	29	77	32	32	8	24	17	17	6	2	1	1	2	1	1	37	3	0.8		0.5	6	10		0.3
MDMA	3.3	14	6	15	6	6	3	4	3	4	4							26				1	6	3		
Azoxystrobin	10.2														0.7	0.8	1	1								
Tamsulosin	8.1	2	0.8	2	1	1		0.8	0.5	0.6	0.4							2	0.4							
Memantine	5.9	14	5	20	8	9	1	4	4	5	0.8	10	5	3	5	4	4	19	3	2				1		
Citalopram	1.6	198	86	273	147	153	47	80	69	74	59							374								
Pirenzipine	1.0																	5								
Carbamazepine 10,11-epoxy	4.2	41	22	70	34	39	20	30	21	22	7	20	10	9	22	14	15	87	20	10		0.5	2	13		
Carbamazepine	12.3	69	35	85	43	50	41	45	31	33	14	41	23	21	44	29	33	121	42	26	0.6	0.7	2	4		
Tramadol	1.9	487	217	711	323	344	180	250	207	240	38	175	76	69	96	62	81	400	116	60		19	69	49		
Ketamine	2.7	489	193	417	176	204	5	137	112	106	20	105	40	33	45	33	36	234	72	20		2	10	15		
Trimethoprim	6.3	38	18	46	27	25	17	20	14	13	5							88					24	5		
Temazepam	8.2	11	6	16	7	8	2	7	5	5	2	3	2	1	4	3	3	16	3	3			1	2		
Terbutryn	6.1	2	1	2	1	2	9	1	1	2	4							3					16	12		
Venlafaxine	1.6	95	36	180	62	68	8	41	28	32	21	61	26	24	31	19	29	279	33	13				71		
Sulfapyridine	6.0	154	75	126	78	79	51	70	45	40	11	43	25	18	37	23	24	213	51	10						

	Sheffield										Norwich										London				
	A	B	C	D	E	F	G	H	I	J	A	B	C	D	E	F	G	H	I	J	A	B	C	D	E
Propranolol																	132								
Acetamiprid																	19	19							
Venlafaxine	45	42	52	53	45	14	52	29	22		30	35	30	42	33	34	276	54	42				50	38	31
Amitriptyline																	13								
Bezafibrate																	83							83	33
Trimethoprim																	19								
Diphenhydramine																	18								
Ketamine	13	11															29	13	13						
Tramadol	12	11	12				12				17	18	14	21	16	18	106	31	24						
Citalopram											14	14		14	14		58	16	18						
Lidocaine																	12						27	13	39
Methamphetamine												22													
Valsartan																									11

Supplementary Figure 3. *The values used to calculate the RQs in Supplementary Figure 2. Bottom: average concentrations measured directly in water (ng L^{-1}), top: the TWA concentrations (ng L^{-1}) derived from the average contaminant mass on the 3D-PSD and the R_s value (mL day^{-1})* [1].

Supplementary Note 1. Participant questionnaires – pre-training

Demographics (Collected via Microsoft Forms)

1. Please tell us with community/kayak/canoe club you are participating with
2. Please enter the first half of your postcode
3. Please tell us your age
4. What gender do you identify as?

River use (Collected via Microsoft Forms, refer to Table S7 for participant responses)

1. How frequently do you visit or utilise your local river? (*Select only one response*)
    a. Daily
    b. 2-3 times a week
    c. 4-5 times a week
    d. Average once every 2 weeks
    e. Monthly
    f. A few times a year
2. In which ways do you spend time in or around the river? (*Select all that apply*)
    a. Kayaking/ Canoeing/ Stand-up paddleboarding
    b. Walking alongside
    c. Swimming
    d. Other, please share _____
3. On average, how much time do you spend per visit to the river? (*Select only one response*)
    a. 1 - 30 minutes
    b. 31 - 60 minutes
    c. 61 - 90 minutes
    d. 90 – 120 minutes
    e. 2 – 4 hours
    f. 4 + hours
4. Who do you bring with you when you spend time at the river? (*Select all that apply*)
    a. Community members
    b. Family
    b. Friends
    c. Partner
    d. Spend time alone

e. Other, please share _____

5. On average, how many people you explore/enjoy your local river with (ie, 1 partner, 3 friends, a school group of 10 or more)? (*Select only one response*)
   a. 0
   b. 1-2
   c. 3-5
   d. 6-9
   e. 10+
6. Which months of the year will you visit the river more than once? (*Select all that apply*)
   a. January
   b. February
   c. March
   d. April
   e. May
   f. June
   g. July
   h. August
   i. September
   j. October
   k. November
   l. December
7. How many years have you been a frequent river user? (*Select only one response*)
   a. 0-3 years
   b. 3-7 years
   c. 7-12 years
   d. 13 + years
8. Which river locations do you visit/utilise most often? (*Open answer*)

Pollution perceptions (Collected via Microsoft Forms, refer to Table S8 for participant responses)

1. The following questions are to be answered regarding your local river or most used/visited river site. Please share with us the name of this river/river location here: (*Open answer*)
2. On a scale of 1-10, with 1 being the 'least knowledgeable' and 10 being the 'most knowledgeable', how knowledgeable do you feel you are regarding your local river's health? (*Open answer*)

3. What are your main sources of knowledge? (*Select all that apply*)
    a. First-hand experience
    b. Publications by non-profits or charities
    c. Local news
    d. Rivers Trust Sewage Map
    e. Scientific journals
    f. Other ____
4. On a scale of 1-10, with 1 being the 'no pollution' and 10 being the 'very polluted', how polluted do you think your local river is? (*Open answer*)
5. How would you define river pollution? (*Open answer)*
6. Are there areas of your local river that you believe are prone to pollution? If so, why? (*Open answer*)
7. Are there areas of your local river which you believe are more clean? Please elaborate whether you answer yes or no. (*Open answer*)
8. Do you have any specific concerns or worries about your local river's health? This may include a specific pollution source, a type of pollutant or certain locations. Please provide as much detail as possible (*Open answer*)
9. Is there anything in particular you would like to learn about your river's health? (*Open answer*)
10. Is there anything else you would like to tell us about your local river? (*Open answer*)

Engagement (collected via Mentimeter, refer to S4 for participant responses)

1. What word comes to mind when you think of your river? (*Open answer*)
2. What contaminants interest you the most? (*Open answer*)
3. What do you think we're going to find in our samples? (*Open answer*)

Supplementary Table 7. Summary of participant responses to the pre-training questionnaire – River use. Answers are expressed as a percentage of the total number of responses per question, and the responses to the open-ended questions are randomised.

1. How frequently do you visit or utilise your local river? (responses: n = 9)

Daily	2-3 times a week	4-5 times a week	Average once every 2 weeks	Monthly	A few times a year
11 %	11 %	33 %	33 %		-

2. In which ways do you spend time in or around the river (responses: n = 18)

Kayaking/ Canoeing/ Stand-up paddleboarding	Walking alongside	Swimming	Other
44 %	44 %	11 %	-

3. On average, how much time do you spend per visit to the river? (responses: n = 9)

1 – 30 minutes	31 – 60 minutes	61 - 90 minutes	90 – 120 minutes	2 – 4 hours	4 + hours
11 %	11 %	33 %	11 %	33 %	-

4. Who do you bring with you when you spend time at the river? (responses: n = 20)

Community members	Family	Friends	Partner	Spend time alone	Other
15 %	15 %	20 %	10 %	25 %	• Youth groups – 5 %<br>• Canoe club – 5 %<br>• Customers – 5 %

5. On average, how many people you explore/enjoy your local river with (ie, 1 partner, 3 friends, a school group of 10 or more)? (responses: n = 9)

0	1-2	3-5	6-9	10+
-	11 %	33 %	22 %	33 %

6. Which months of the year will you visit the river more than once? (responses: n = 102)

January	February	March	April	May	June
7.8 %	7.8 %	8.8 %	8.8 %	8.8 %	7.8 %
**July**	**August**	**September**	**October**	**November**	**December**
7.8 %	7.8 %	8.8 %	8.8 %	8.8 %	7.8 %

7. How many years have you been a frequent river user? (responses: n = 9)

0 – 3 years	3 – 7 years	7 – 12 years	13 + years
-	22 %	22 %	55

8. Which river locations do you visit/utilise most often? (*Open answer*)

- River Don
- River Derwent
- Don
- Sheaf
- Porter Brook
- Thames in Central London
- Kew
- Richmond
- Berkshire
- River Wensum and River Yare
- Paddling down towards Surlingham
- Mainly Welsh rivers and northern England rivers.
- River Don (Sheffield)
- River Derwent (Matlock, Darley Dale)
- River Calder (Sowerby Bridge, Calderdale)
- River Yare
- River Wensum
- River Wensum/Yar
- River Bure
- River Don - Sheffield (SCKC activity)
- River Derwent - Derbyshire (River Guide)
- River Dee - Llangollen
- River Tryweryn - Bala
- River Tees - Teeside
- River Dart - Dartmouth
- River Leven - Lake District

Supplementary Table 8. Summary of participant responses to the pre-training questionnaire – Pollution perceptions. Answers are expressed as a percentage of the total number of responses per question, and the responses to the open-ended questions are randomised.

1. The following questions are to be answered regarding your local river or most used/visited river site. Please share with us the name of this river/river location here: (*Open answer*)

- River Don
- River Don
- River Yare and River Wensum
- Don
- River Yare
- River Don
- Don
- River Wensum
- Thames in Central London

2. On a scale of 1-10, with 1 being the 'least knowledgeable' and 10 being the 'most knowledgeable', how knowledgeable do you feel you are regarding your local river's health? (*Open answer*)

- 7
- 6
- 4
- 5
- 4
- 4
- 5
- 7
- 4

3. What are your main sources of knowledge? (responses: n = 17)

First-hand experience	Publications by non-profits or charities	Local news	Rivers Trust Sewage Map	Scientific journals	Other
47 %	5.8 %	29 %	5.8 %	-	• SWSG and Upper Don Trail Trust – 5.8 %<br>• BBC online – 5.8 %

4. On a scale of 1-10, with 1 being the 'no pollution' and 10 being the 'very polluted', how polluted do you think your local river is? (*Open answer*)

- 6
- 7
- 6
- 7
- 4
- 5
- 8
- 7
- 4

5. How would you define river pollution? (*Open answer*)

- Combined sewer out flow, surface run off and ocassional industrial pollution.
- Any manmade or unnatural waste/product in the river, in particular sewage and pollution leaching from ex industrial land
- Contamination of the river
- Anthropogenic impacts
- The contamination of water sources by substances or materials.
- Pollution from CSO's - untreated sewerage
- Dumping of man made object in the river (car parts/plastic bottles etc)
- Presence of substances, chemical and biological harmful to the ecosystem and to human health
- Non natural substances and debris
- Contamination of the river, such as through sewage and agricultural/urban runoff. Presence of toxic substances (oil, pesticides, plastics, waste). Anything that changes water - such as it's PH, salinity and temperature.

6. Are there areas of your local river that you believe are prone to pollution? If so, why? (*Open answer*)

- CSO locations, sewage works and industrial/vehicule repair locations. Waste oil entering river is infrequent but extremely impactful when it does happen.
- Yes - outfalls observed and unpleasant smells
- Sewage treatment nearby sometimes overflow, and often strong smells from the area.
- Waste from factories in city.
- Adjacent to CSO's
- The whole river is affected by old sewage facilities upstream at Deepcar and Oxspring and potentially the new facility in Wharncliffe Side. The old forges and mills are also likely hotspots
- thorpe island posible sewerage leaks the river was covered in a layer of very thick weed this summer making it difficult and dangerous to paddle that stretch.
- yes the river wensum through the city centre new mills to riverside. overflows from drains going into the river
- river yare near trowse mill overflow of sewerage after heavy rain. near postwick bridge river yare outflow from water treatment plant strong smell of chemicals and contamiation paper etc seen in the water.
- Don’t know

Supplementary Table 8. Cont.

<table>
<tr><th colspan="3">7. Are there areas of your local river which you believe are more clean? Please elaborate whether you answer yes or no. (Open answer)</th></tr>
<tr>
<td>No generally. There may be cleaner sections a few miles upstream of the City. In the city area the pollution levels vary more by time than location. For example, a day or so following heavy rain after a period of little rain results in significant street run off into the river. Periods of time when the CSOs are running, again usually during/following high rain. etc.</td>
<td>Yes - away from the city
Closer to the source upstream of industry.
I would guess most of the local rivers are not very clean as the river is tidal the polution travels.
The upper sections of the River Don above Deepcar is a less populated area as the river here is closer to the source and further away from populated areas</td>
<td>No, not in the area I use. Upstream of Pensitone the river is still tinted brown but more from peat discolouration. The rest of the Don is often brown and murky.
Sections of the river before it enters the city likely cleaner.</td>
</tr>
<tr><th colspan="3">8. Do you have any specific concerns or worries about your local river's health? This may include a specific pollution source, a type of pollutant or certain locations. Please provide as much detail as possible (Open answer)</th></tr>
<tr>
<td>Combined Sewer Outflow seems to be an increasing issue. They are running more often and seem to be more polluting than ever before.
Word of Mouth - impacts of local sewage inputs
All combined sewer overflows locations along the river. We sometimes have large volumes of dead fish floating in the river. The smell of sewage is at times very stong.
Also the impact industry has on the health of the river as it passes through Industrial Sheffield.
Is agricultural run off a cause for concern in therm of the health of the river and to human activity in/on the river.</td>
<td>The ex industrial sites are in places being ameliorated by new development so should in theory be improving the sewage however has got worse and results in stomach complaints amongst club members.
I know that industry only really abide by ph levels to monitor effluent which doesn’t eliminate trace amounts of chemical.
Water often appears murky and strong-smelling.
Speeding boats increasing the erosion of the riverbank.
Mainly overflow and sewerage releases during storm events.</td>
<td>Yes thorpe island possible leak from sewerage pipes early summer, the river near there was covered in thick weed making it difficult and dangerous and difficult to paddle.
Trowse mill overflow of sewerage seen in the river near the club occasionally after heavy rain
Often oil (I suspect from powerboats/hire cruisers) seen on surface of river, most frequently in summer.
Waste (such as cans) from tourists often higher in summer.
Runoff from farms and waste from factories.</td>
</tr>
<tr><th colspan="3">9. Is there anything in particular you would like to learn about your river's health? (Open answer)</th></tr>
<tr>
<td>Pollution levels over time and sewage levels.
I would like to know if more detail about what pollutants are in the river but also if there are cleaner areas. It would also be good to understand if the pollutants are something that can be improved.</td>
<td>sewage levels
What is being done to enforce and tackle spills, who is monitoring and taking action.
yes i would like to learn about how clean the river is and how safe it is to swim in.</td>
<td>Yes how much pollution is present and it’s type.
Levels of pollution.
Presence of diseases.
Changes over time.
Health of the river in therms of ecosystem and supporting nature
Risk to human health</td>
</tr>
<tr><th colspan="3">10. Is there anything else you would like to tell us about your local river? (Open answer)</th></tr>
<tr>
<td colspan="2">The river used to be devoid of fish and predatory birds such as herons and kingfisher. These ahve returned so it is important to appreciate how far the river has come in the last 30 years.</td>
<td>Lot's of wildlife, inc. birds and fish. Often frequented by people, mostly in the summer.</td>
</tr>
</table>

Supplementary Note 2. Participant responses to pre-training Mentimeter questions

2a. Sheffield

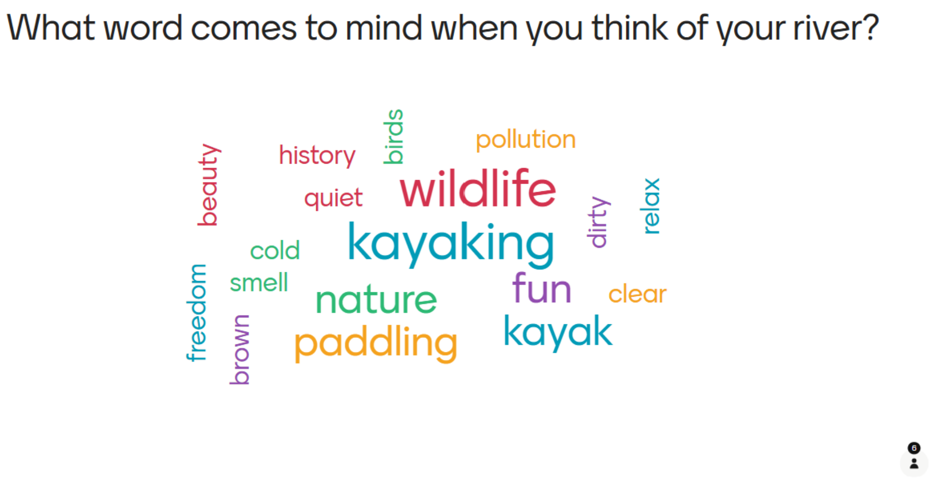

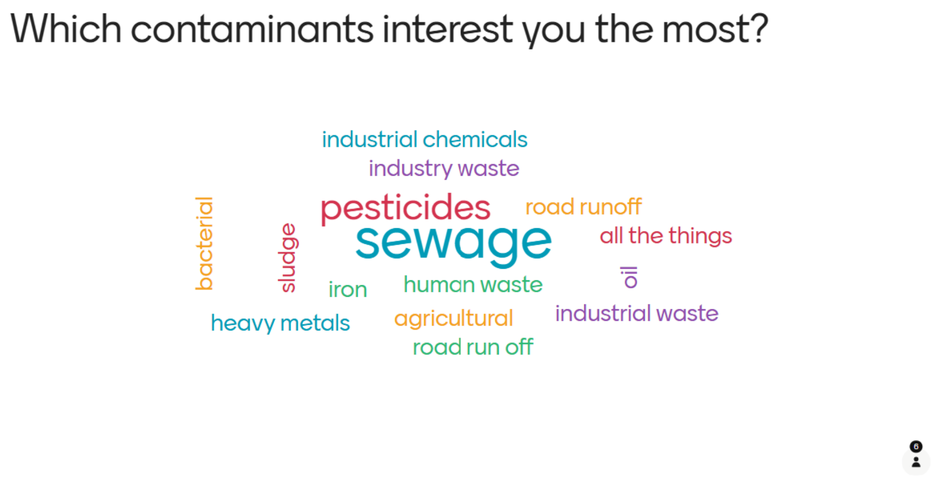

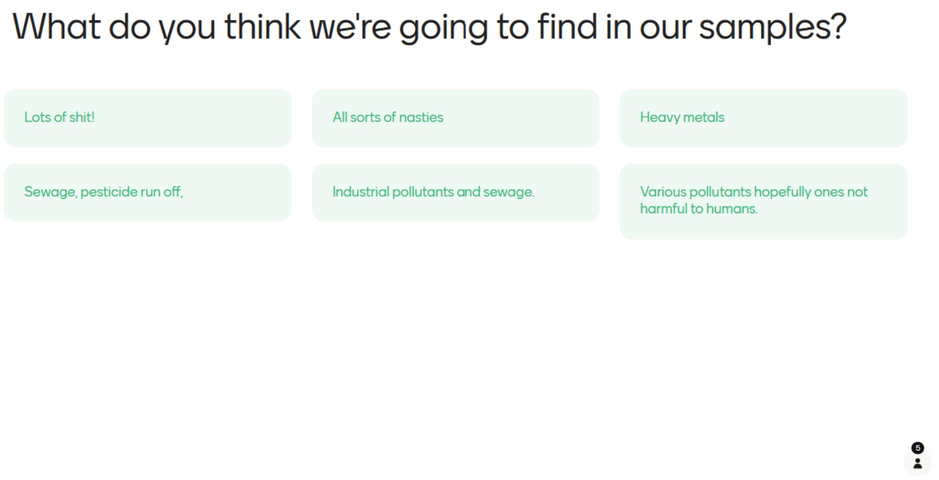

2b. Norwich

What word comes to mind when you think of your river?

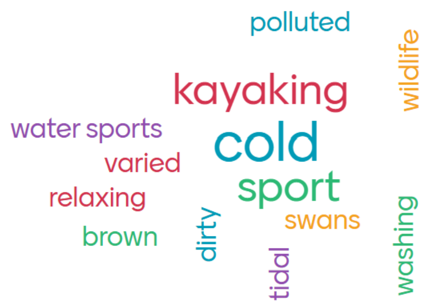

Which contaminants interest you the most?

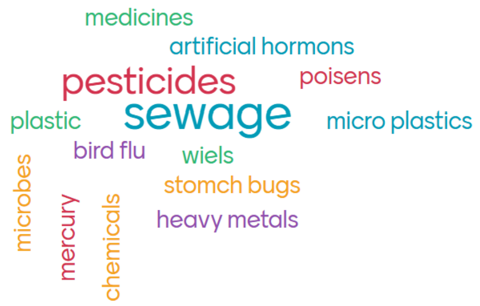

What do you think we're going to find in our samples?

Pesticides from farms	Sewage and molecules from city waste water	Strong concentrations of chemicals
Sewage, chemical run off, oils, diesel,	Chemical and sewage	Unfiltered molecules from waste
Cleaner upstream than downstream (and past sewage works)	Tas cleaner than Wensum (though maybe more pesticides)	More of most things in the city than above. Lots down stream of main sewage plant and upstream in tidal reach. Much less at Caister and cotessey.

2c. London

What word comes to mind when you think of your river?

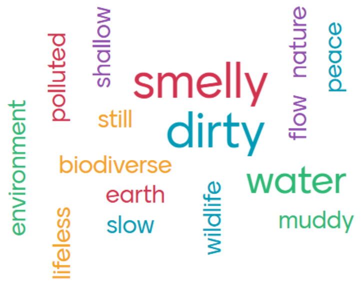

Which contaminants interest you the most?

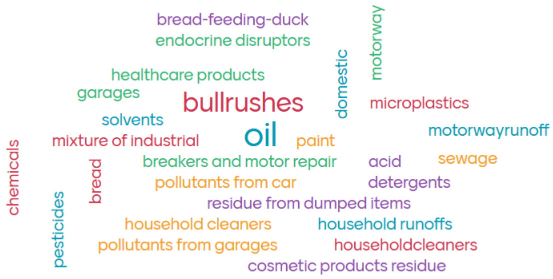

What do you think we're going to find in our samples?

- Mixture of industrial and domestic waste products
- residue from items dumped in the waterway
- Industrial waste
- Home use pharmacy product residues
- Herbicides/pesticides from gardens near the river.
- Pollutants from car shampoo, sewage, engine oil, pesticides, pharmaceutical and cosmetic products, car paint, household cleaners, possibly narcotics...

Supplementary Note 3. Participant questionnaires – post-sampling

Method feedback (collected via Microsoft Forms, refer to Table S9 for participant responses)

1. Please tell us with community/kayak/canoe club you are participating with (*Open answer*)
2. What methods did you use to deploy your passive samplers (rope/weight/zip ties, etc.)? (*Open answer*)
3. How did deployment/retrieval of samplers go? What did you like about it? What was challenging? (*Open answer*)
4. Anything else you'd like to share with us? (*Open answer*)

Results session feedback (collected via Mentimeter, refer to S5 for participant responses)

1. Did anything surprise you about our findings? (*Open answer*)
2. What is your biggest take home message from this project? (*Open answer*)
3. What more would you like to learn about your rivers? (*Open answer*)
4. Would you participate in an updates version of the IMPART project? (*Open answer*)

Supplementary Table 9. Summary of participant responses to the post-sampling questionnaire. The responses to the open-ended questions are randomised.

1. Please tell us with community/kayak/canoe club you are participating with (*Open answer*)

- Thames21
- Norwich Canoe Club
- Norwich Canoe Club
- Sheffield Canoe Club
- Friends of Stoneyfields Park
- Norwich Canoe Club
- Norwich Canoe Club
- Sheffield
- Friends of Stonyfields Park
- Sheffield City Kayak Club
- Sheffield Canoe Club
- Sheffield City Kayak Club
- Norwich Canoe Club
- Silkstreamfriends-Thames21
- Sheffield City Kayak Club

2. What methods did you use to deploy your passive samplers (rope/weight/zip ties, etc.)? (*Open answer*)

- "A whole mixture :-)
- The river Don north of Sheffield is quite stony. So we only pinned the samplers to the river bed once. The rest of the time it was tied to bricks, metal rods, large stones etc"
- Weight and line method
- We used rope/weight/zip ties and pins in the stream at 2 locations and at the other 2 locations we attached the samplers with a rope to a slightly overhanging branch.
- NA - Volunteer Coordinator
- string and wight in most sites. Stappled in bed in two sites.
- "Weights, rope and zip ties x3
- Garden hose stakes and zip ties x2"
- We used weights attached to staples or string. The string was tied to various things, such as bricks, metal debris or solid objects on the bank. All depending on what was available.
- Rope on one/ other two metal loops
- Rope and weight tied on to object
- Ropes and weights
- "2 with Pegs
- 1 with rope and weight"
- "Floaters and weight and zip ties
- 3 of the 4 were secured to the bank using a peg. One was tied to a drainage gate"
- Rope, weight & zip ties
- Weight and floats mainly

3. How did deployment/retrieval of samplers go? What did you like about it? What was challenging? (*Open answer*)

- Working in the kayak can be hard. Another and I used a k2 (2man kayak) one person holding us steady other doing the samples. In a k1 (sigle kayak) you would need to get out. Putting on gloves with wet cold hands took most of the time, with carfully handing or a change in sensor deisgn maybe it would be possible not to use gloves?
- Paper and pens combined with water does not work very well trying to note down sample number, times etc. The key I think would be to make sure as much of the paperwork is completed before you setoff or maybe some simple sofware and barcodes with a phone. same goes for rope and lines etc getting the kit pre made up on the land might have made the process quicker eaiser than at location (maybe have the rope and weight cut to a longer length and mounted, so you fit the sensor and remove unneeded rope length when you get to location). The time of the year to take samples needs to be considered currenty we are having large tide chanages, other times of the year the river height is more stable. Making sure the samples are put out and collected at low tide is important, other wise there is a risk the sample will not be fully under the water at all time.
- I only deployed but this was very successful. We organised ourselves to have small bags on equipment for each sampler. Work very well with three people each having a job.
- "We found locations quickly and placed samplers.
- Took photos and w3w of locations but didn't use the red coloured markers so took a while searching for the exact position of the samplers on return."
- No issues. All retrieved fine. One of the samplers had gathered a significant amount of dead leaves and branches which appeared to have weighted it down. Notes recorded on the results sheet.
- We misjudged how long each site would take. The combination of paddling to the sites, messing about at the sites and paddling to next etc.... all in 24hours was too much exercise ontop of our normal training programmes. Retrieval was a little quicker. Other comments I have already put in emails
- Fairly easy and fun to do. Took a little longer than first thought. Slightly tricky trying to put on gloves with wet hands! Day of retrieval was very windy, so trying to keep all bags, etc. in place was challenging. Had a couple of people in the more public spots and other boat clubs inquire about the project.
- In our river it was really fun. I and the person I deployed the samplers with know the river well, are fairly fit and fairly adventurous. Slippy rocks, spiky brambles and loose banks add to the fun. *but* I suspect others would have found it physically challenging. A bit of local knowledge and confidence helped a lot. Working in a pair was really important; one making notes whilst the other gets wet is part of it, but also the safety element of if something went slightly wrong (some of the places we deployed samplers probably haven't seen people in weeks and had poor mobile signal) means it was important we had someone else there. Just to be clear; we weren't exactly abseiling off cliff edges or taking any massive risks, but it only takes a relatively small turn of the ankle at the bottom of a river bank to be in trouble.
- Ok
- The deployment was done in the rain, retrieval was a nice sunny day. went well took a bit longer than I thought It would. It was interesting going to different places on the river,
- The area at the 4th location next to the Silkstream trash screen was rather muddy/slippery when we collected the sampler, but it went smoothly.

Supplementary Table 9 cont.

• The river height must have changed over a a couple of foot in time paddling putting out sensors. Make sure you have enough time so you are not rushing from place to place (but its good training) and maybe try and avoid heavy rain so you can to enjoy the experince and not freezze... lol	• Enjoyed the detailed nature of records, accessing the river in new places and setting up each method of fixing.<br>• Deployment- not doing it systematically, first one we did forgot at first to do air sample, went into water without the water sample bottle. Thought to much about putting them somewhere safe on Retrieval ended up making it difficult not easy to retrieve knee high in mud nearly losing wellies	• "It went well. We chose 4 locations spread out in the catchment. One of the locations along the Silkstream at the end of Market Lane near garages from where we suspect polluted water from spray jobs and cars being washed is seeping into the stream plus very littered and people with alcohol and substance related issues congregating there. We knew by starting early we knew it would most likely be quiet and safe to place and collect the sample. At another location downstream from where tributary ditches join the stream we waded through the stream to placed the sampler. We brought safety wellies and a wading pole for stability.<br>• The area at the 4th location next to the Silkstream trash screen was rather muddy/slippery when we collected the sampler, but it went smoothly.<br>• Deployment and retrieval by canoe was good but reasonably challenging in low water, but allowed us to access areas otherwise impractical on foot.
	4. Anything else you'd like to share with us? (*Open answer*)	
• The bobble floats weren't used because we didn't want to attract attention to the sampler but we did use the green tape attached to the brick or weight which was fine once within a couple of metres. The river at the north end is shallow, less than knee deep in most places so this worked for us.<br>• No matter how you deploy your samplers we would recommend that you secure orange floats to them all. when you tie them to the metal loops it will make it so easy to find your samplers especially as you have pushed the loops into the ground you have little idea where they are even though you took photos<br>• Went fine<br>• Really enjoyed putting out the samples, looking forward to the results.	• I've lived in Sheffield 20+ years and near the river 10+ years. I'd never really thought about how much we interact with and engage with the water system arround us as a family and as individuals. It was also really eye-opening to think about just how many places the river has yuck and grot flowing into it; be it the obvious stuff like the water works and factories or the less obvious stuff like leaching in from building sites and roads.<br>• We found it best to put bright tape about 30cm below the water on the rope, so easy to find when you're looking for it, but passers by wouldn't see it. Definitely best to take photos of where the sampler was deployed, as it makes retrieval much easier.	• If you made a short video showing how to prepare deploy and collect samplers that could be used in future projects. Then could be used in training, and by participants in the field as reminder. I would be happy to participate in this if you wished.<br>• The deployment method instructions seemed to be overly complex. They could be streamed lined and made into an easier to follow flow diagram which might be easier for non-technical people to follow. The bag then double bag instruction seemed a tad contradictory. The box to return the samples needed to be a bit bigger to fit more ice blocks in.<br>• Enjoyed taking part. I am interesting to hear the results

Supplementary Note 4. Participant responses to the Mentimeter questions at the results Zoom meeting in November of 2022.

Did anything surprise you about our findings?

- The pesticides are all banned
- The variation in chemicals at the different sites, the 'chemical fingerprint'
- I was expecting the city results to be worse than the upstream rural parts of the e river.
- Slightly surprised the the readings at the peak waste overflow diluted so quickly. The volume of chemical and types are interesting.
- Sheffield seemed worse than others and not that much better than the worse polluted river in London
- how much the pollutants dilute going downstream
- .
- I honestly thought that with a river that flows fairly day and looks clean (river don) just hope many chemicals we're found and in high concentrations
- That the caister site on river Tas Norwich was high suprised me
- The high results of carbamazepine particularly in Norwich.

What is your biggest take home message from this project?

- There are alot of things in the water that you wouldn't expect
- I need more time to take in the information/data which you just presented
- Can you measure PFAS using your 3D Printed discs?
- More work needs doing
- Passive samplers give you a much better idea of ecological impact compared to spot samples. Do we have PNECs for pharmaceuticals?
- I never would have considered pet products would ending up in the river in such large quantities
- Don't take our river for granted, it looks clean but there's so much more to do
- There's more work to be done to change people's attitudes to chemical use and disposal
- Lots more work is required in the waste treatment plants to remove more chemical content.

What more would you like to learn about your rivers?

What do I need to know to use the rivers safely?

I'd love to know how the river changes over time

I think it has to include microbial analysis to cover all sides of pollution

Chemical profiles change over time and between seasons. It would be really helpful to get a seasonal profile for these substances.

It would be interesting to get a regular general update about the ecological state of the local waterways, the issues/challenges/improvements...

Do some of these chemicals get concentrated up food chain? Are our otters at risk? Could sampling otter scatts answer that?

Can you detect more than 200 substances? The EA use semi quantitative analysis for over 1500 compounds and NRW use passives but analysis is done for over 2300 compounds

How the chemicals can affect river users. More samples at difference locations and key sites across the year.

7

Would you participate in an updated version of the IMPART project?

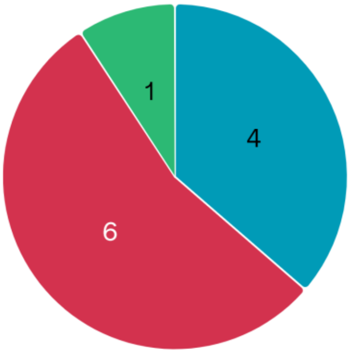

4	Yes, as a first-timer.
6	Yes, for the second time!
1	No, not for me.

11

Supplementary Note 5 – List of analytical standards used

Reference standards for 2-(thiocyanomethylthio)benzothiazole, 4-fluoromethacationone, 4-methylethcathinone, acetamiprid, aclonifen, alprazolam, ametryn, amiodarone, amitriptyline, amlodipine, amphetamine, antipyrine, atorvastatin, atrazine, azelnidipine, azithromycin, azoxystrobin, benoxacor, bensulide, benzatropine, benzoylecgonine, betaxolol, bezafibrate, bisoprolol, bupropion, buspirone, carazolol, carbamazepine, carbamazepine-10, 11-epoxide, carboxine, carfentrazone-ethyl, celecoxib, chloramphenicol, cilazapril, citalopram, clarithromycin, clodinafop-propargyl, clofibric acid, clopidogrel, clothianidin, clozapine, cocaine, cyclouron, cycloxyidim, cymoxanil, cyphenothrin, cyromazine, diazepam, diclofenac, diflubenzuron, dimethametryn, dimethomorph, diphenhydramine, disulfoton sulfone, diuron, enalapril, ethofumesate, famoxadone, fenoxaprop-ethyl, fenuron, flufenoxuron, fluocinonide, fluoxetine, flurbiprofen, flurochloridone, flutamide, flutolanil, fuberidazole, gemfibrozil, haloperidol, hydrochlorothiazide, ibuprofen, imidacloprid, indomethacin, isocarbamid, isradipine, josamycin, ketamine, ketoconazole, ketoprofen, ketotifen, levamisole, levocabastine, lidocaine, lincomycin, lorazepam, MDMA, meclizine, meclofenamic acid, medroxyprogesterone, mefenamic acid, memantine, mephedrone, mephosfolan, metformin, methamphetamine, methcathinone, methedrone, methylphenidate, metoprolol, morphine, nadolol, naproxen, nicotine, nifedipine, nitenpyram, nordiazepam, norethisterone, nortriptyline, orphenadrine, oxamyl, oxazepam, oxycarboxin, oxycodone, picoxystrobin, piperophos, pirenzipine, pretilachlor, prodiamine, prometon, prometryn, propamocarb, propranolol, propazine, pymetrozine, pyracarbolid, pyraclostrobin, pyraflufen-ethyl, pyridaben, risperidone, rizatriptan, ronidazole, roxithromycin, salbutamol, salicylic acid, sertraline, simazine, spinosyn A, spinosyn B, spiramycin, sulfadimethoxine, sulfamerazine, sulfamethazine, sulfamethoxazole, sulfamonomethoxine, sulfapyridine, sulfathiazole, sulfisoxazole, tacrine, tamsulosin, temazepam, terbutryn, terfenadine, thiacloprid, thiamethoxam, thiazopyr, timolol, tramadol, trimethoprim, valsartan, venlafaxine, verapamil, warfarin and ziprasidone were sourced from QMX (Essex, UK).

Deuterated internal standards for amitriptyline-d3, amphetamine-d6, benzoylecgonine-d3, betaxolol-d7, celecoxib-d7, cetrizine-d4, clarithromycin-d3, clothianidin-d3, cocaine-d3, cotinine-d3, diazepam-d6, fluoxetine-d6, gemfibrozil-d6, haloperidol-d4, ketamine-d4, lidocaine-d10, lorazepam-d4, MDMA-d5, methylone-d3, methylphenidate-d9, metoprolol-d7, morphine-d3, nicotine-d4, nifedipine-d4, nordiazempam-d5, nortriptyline-d3, oxazepam-d5, risperidone-d4, sertraline-d3, sulfamethazine-d4, temazepam-d5, thiamethoxam-d3, tramadol-d3, trimethoprim-d3, venlafaxine-d6 and verapamil-d3 were purchased from Sigma Aldrich (Gillingham, Dorset, UK) and QMX (Essex, UK).

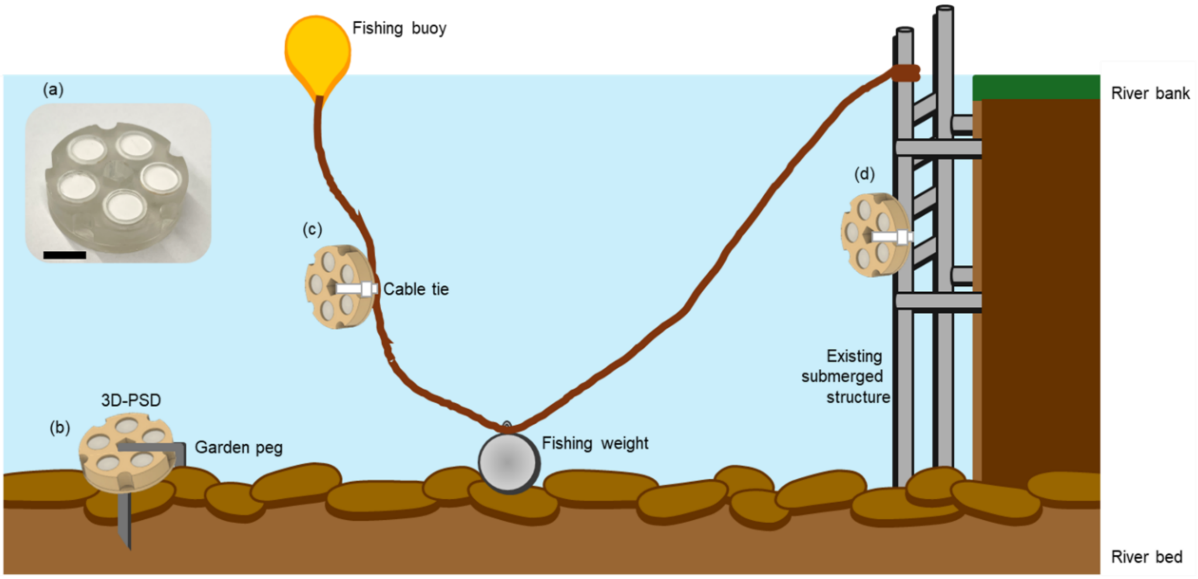

Supplementary Figure 4. *(a) Assembled 3D-PSD ready for deployment, black scale bar represents 9 mm. Illustrations of the different deployment methods demonstrated to the citizen scientists during the training session, not to scale; (b) 3D-PSD fixed to the river bed using a U-shaped garden peg, (c) 3D-PSD cable tied to a rope weighed down with a fishing weight and anchored to small buoy or the river bank, (d) 3D-PSD cable tied to an existing submerged structure.*

Supplementary Note 6 – Recruitment flyer sent to the community groups. Design created in Canva

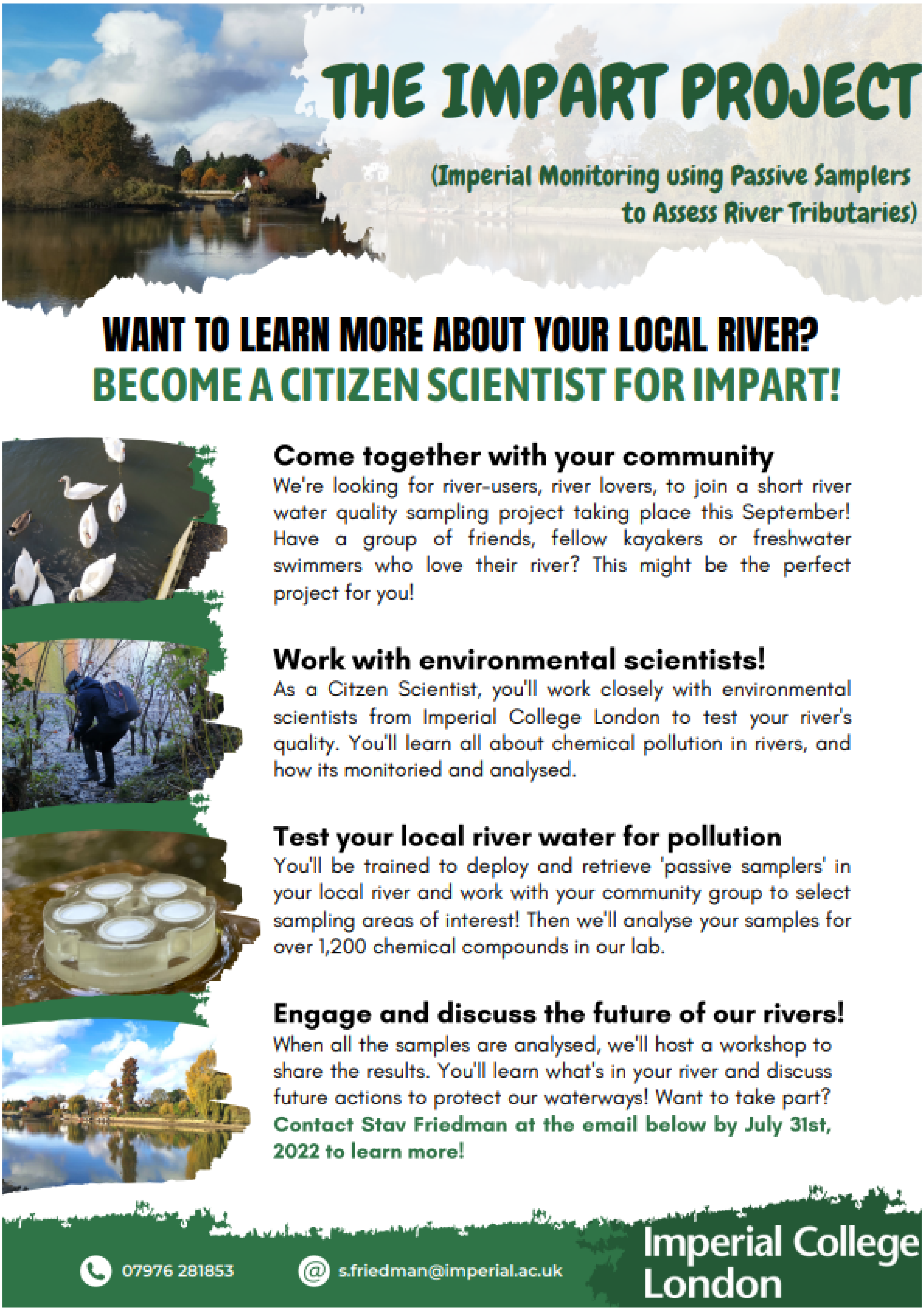

Supplementary Note 7 - Sampling equipment provided to the citizen scientists

- 3D-PSDs for deployment
- Field blank 3D-PSDs
- Transport blank 3D-PSD
- 30 mL Nalgene bottles
- Roll of biodegradable flagging tape
- Fishing weights (300 g)
- Fishing buoys
- U-shaped garden pegs
- Cable ties
- Paracord
- Gloves (variety of sizes)
- Waste bags for the gloves
- Resealable plastic bags
- MeOH-washed aluminium foil
- Polystyrene cooler box
- Reusable ice packs
- Packing tape
- Permanent markers
- Data sheets
- Sampling protocols

Supplementary Table 10. Contaminant concentration on the PES membrane and HLB sobent (ng) after exposure. Of the 164 exposure contaminants, only 14 compounds were quantified on the PES membrane after 7 days over the range of 0.004 ± 0.001 (timolol) to 0.2 ± 0.09 ng (propazine). Representing, on average, less than 30 % of the contaminant mass accumulated on the HLB disk after 7 days.

Compound	LOD	LLOQ	[PES] t=7d	[HLB] t=7d
Acetamiprid	0.002	0.01	0.2 ± 0.04	1.9 ± 0.6
Betaxolol	0.01	0.02	0.06 ± 0.03	0.8 ± 0.7
Bisoprolol	0.002	0.01	0.02 ± 0.02	0.7 ± 0.5
Bupropion	0.01	0.03	0.07 ± 0.05	0.07 ± 0.05
Carazolol	0.001	0.004	0.1 ± 0.03	0.9 ± 0.6
Carbamazepine	0.01	0.02	0.02 ± 0.01	1.9 ± 0.6
Ketamine	0.01	0.02	0.03 ± 0.01	0.5 ± 0.4
Ketotifen	0.02	0.05	0.06 ± 0.01	0.6 ± 0.8
Lidocaine	0.001	0.003	0.008 ± 0.004	0.6 ± 0.8
Methedrone	0.005	0.01	0.04 ± 0.02	0.02 ± 0.01
Nordiazepam	0.01	0.03	0.1 ± 0.05	1.4 ± 0.6
Oxazepam	0.01	0.02	0.07 ± 0.01	1.9 ± 0.7
Tacrine	0.01	0.02	0.05 ± 0.02	2.0 ± 0.7
Timolol	0.001	0.002	0.004 ± 0.001	1.1 ± 0.7

Supplementary References

1. Richardson, A. K. *et al.* A miniaturized passive sampling-based workflow for monitoring chemicals of emerging concern in water. *Science of The Total Environment* **839**, 156260 (2022).

2. NORMAN Ecotoxicology Database. https://www.norman-network.com/nds/ecotox/lowestPnecsIndex.php (2024).
